# Ecosystem-level carbon storage and its links to diversity, structural and environmental drivers in tropical forests of Western Ghats, India

**DOI:** 10.1038/s41598-020-70313-6

**Published:** 2020-08-10

**Authors:** Subashree Kothandaraman, Javid Ahmad Dar, Somaiah Sundarapandian, Selvadurai Dayanandan, Mohammed Latif Khan

**Affiliations:** 1 Department of Ecology and Environmental Sciences, Pondicherry University, Puducherry, 605014 India; 2 Biology Department, Centre for Structural and Functional Genomics, Concordia University, 7141 Sherbrooke St. W., Montreal, QC H4B 1R6 Canada; 3 Department of Botany, Forest Ecology and Eco-Genomics Laboratory, Dr. Harisingh Gour Vishwavidyalaya (A Central University), Sagar, MP 470003 India

**Keywords:** Ecology, Plant sciences, Ecology, Environmental sciences

## Abstract

Tropical forests are rich in biodiversity with great potential for carbon (C) storage. We estimated ecosystem-level C stock using data from 70 forest plots in three major forest types: tropical dry deciduous (TDD I and TDD II), tropical semi-evergreen (TSE I and TSE II) and tropical evergreen forests (TEF I, TEF II and TEF III) of Kanyakumari Wildlife Sanctuary, Western Ghats, India. The average C stock in these forests was 336.8 Mg C/ha, of which 231.3, 3.0, 2.4, 15.2 and 84.9 Mg C/ha were stored in woody vegetation, understorey, litter, deadwood and soil respectively. The live vegetation, detritus and soil contributed 65.5%, 5.5% and 29% respectively to the total ecosystem-level C stock and distributed in forest types in the order: TEF III > TEF II > TEF I > TSE I > TDD II > TSE II > TDD I. The plant diversity, structural attributes and environmental factors showed significant positive correlations with C stocks and accounted for 6.7, 77.2 and 16% of variance. These findings indicate that the tropical forests in the Western Ghats store large amount of C, and resulting data are invaluable for planning and monitoring forest conservation and management programs to enhance C storage in tropical forests.

## Introduction

The increasing levels of carbon dioxide (CO_2_) in the atmosphere, currently at 416 ppm^1^ remains as a major contributor to global warming and storage of carbon (C) in different pools of forest ecosystems plays a crucial role in combating global warming and mitigating adverse effects of climate change. The current C stocks in the world’s forests are estimated to be 861 ± 66 Pg C of which 42, 8, 5 and 44% are locked in living biomass, deadwood, litter and mineral soil respectively^2^, and the total amount of stored C in the forest vegetation has been estimated to be approximately 359 billion tonnes^3^. Soil is also a major terrestrial C sink, which consists about two-thirds of the total terrestrial C pool^4^. Overall, the amount of C stored in forest ecosystems is twice than that of the atmosphere^5^.

Tropical forests are global centres of biodiversity which play an important role in the regulation of the global and regional C cycles^6–8^. Tropical forests fix 3,040 g C/m^2^/year^9^ and serve as a major potential sink to store C. The high C stocking potential of tropical forests could be attributable to its high species diversity^10^ and high net primary production^11^. Tropical forests store 56% of C in biomass and 32% in soil^2^. A great variation exists among the biomass and C stock estimates for different types of tropical forests (170.3–689.7 Mg/ha)^12^. These wide differences in C stock estimates may be due to variations in tree species richness, stand structural attributes, climatic differences, forest type, altitudinal variations and soil types^13–15^. Understanding the relationship between biodiversity and C storage in these ecosystems^7^ is crucial for devising effective strategies for biodiversity conservation and storage of C to mitigate global warming and climate change.

Tree species richness strongly affects aboveground and belowground C storage^16^, litter production and decomposition^17^ and soil C^18^. Species richness is known to enhance biomass and C stocks of forests^8^. However, the diversity-C relationships are scale-dependent and therefore conservation schemes that are mainly focussed on C storage may fail to protect many species that occur in species-rich tropical forests^19,20^. The diversity-C correlations in tropics at 1 ha scale were absent and within continents are either weak (Asia) or absent (Amazonia and Africa)^7^. In addition to species richness, forest C stocks are highly influenced by many stand structural variables, such as tree size and stand characteristics^21^. Assessment of C stocks in relation with tree species richness is essential for the effective management of tropical forests in a manner that would result in C emission reductions as well as biodiversity conservation as recognized by the Reducing Emissions from Deforestation and forest Degradation (REDD+) scheme, and understanding the relationship between species richness and C stocks is important for effective implementation of REDD+ policies. Biodiversity should not be recognized only as a co-benefit, rather it should be considered mandatory for both short- and long-term maintenance and management of C stocks^22^. Understanding the biodiversity and C stock distributions and potential C-biodiversity trade-offs is essential, as protecting some forests could possibly divert threats to other unprotected areas^7^.

The complex relationship between biodiversity crisis and climate change in tropical forests requires coordinated efforts for effective tackling^20^ of both crises together. Under current climate model scenarios, there is room for establishing an additional 0.9 billion hectares of woodlands and forests which could store about 205 Gt C^23^. Thus, forest restoration serves as a most effective strategy for biodiversity conservation and climate change mitigation^24^. The United Nations Environment Assembly in Nairobi, Kenya declared the decade of 2021–2030 as the UN Decade on Ecosystem Restoration. It is timely to assess the biodiversity and C stocks in biodiversity-rich and ecologically valuable yet understudied ecosystems such as Western Ghats and explore the relationships between C storage and biotic (diversity and structural attributes) and abiotic (environmental factors) factors to formulate effective ecosystem restoration schemes while conserving biological diversity.

Most previous studies focused only on the aboveground biomass C (AGBC) stocks in tree species, without accounting for the roles played by C stocks of understorey (shrubs and herbs), roots, deadwood, litter, soil and small diameter {≥ 3 cm to < 10 cm DBH (diameter at breast height)} tree individuals in tropical forest ecosystems and their relationships with diversity, structural attributes and environmental factors. Also, many such studies explored relationships between C stocks and species richness by either compilation of datasets from other studies or from metadata^7,8^. Assessment of C stocks and their allocation in different ecosystem components is essential to understand the degree to which C is partitioned between labile and stable components^25^. Diversity, structural attributes and environmental conditions have been reported to be strongly associated with C stocks^8,26–31^.

India ranks fourth among countries with highest CO_2_ emissions^32^. However, India also constitutes a forest cover of 7,12,249 km^2^ with a substantial C storage potential and the total C stock of Indian forests is 7,124.6 million tonnes^33^. Nevertheless, these figures are based on not just natural forests, but could also include tree plantations of mostly exotic species^34^. Very few studies have been conducted on the C stock assessments of natural forests in the Western Ghats. Seen et al*.*^35^ found that despite being under pressures such as deforestation and land-cover changes, the forests of Western Ghats maintained ~ 0.43 Pg of soil organic carbon (SOC) stocks. Osuri et al*.*^36^ observed that even large and well-protected forest fragments in Western Ghats store 40% less AGBC than the contiguous forests due to alterations in stand structure, tree allometry and compositional shifts. Padmakumar et al*.*^37^ assessed the tree C stocks of Chinnar Wildlife Sanctuary in Western Ghats and observed a strong positive relationship with basal area. There is a paucity of information regarding the ecosystem-level C stocks and their relationships with diversity {species richness, species evenness, Shannon’s diversity index and Simpson’s dominance index}, structural attributes {density, basal area, big-sized trees (≥ 60 cm DBH) and mean tree DBH} and environmental factors {elevation, mean annual precipitation (MAP), mean annual temperature (MAT), slope and aspect} in tropical forests of India, particularly in Western Ghats. Understanding of such relationships provides us means to gain insights into the relative importance of the different stand structural attributes in ecosystem C storage. Moreover, these relationships also shed light on how the stand structural attributes influence the C partitioning among different ecosystem components (trees, woody climbers, bamboo and palms, understorey, litter, deadwood, and soil) in different forest types of Western Ghats. This will be invaluable for designing frameworks to maintain C stocks in tropical forests and conservation of biodiversity. Therefore, we carried out the present study to (1) estimate the biomass and C storage of total woody vegetation (adults, juveniles, woody climbers, bamboo and palms), understorey, litter, deadwood and soil in three major forest types and seven study sites {tropical dry deciduous (TDD I and TDD II), tropical semi-evergreen (TSE I and TSE II) and tropical evergreen (TEF I, TEF II and TEF III)} of Kanyakumari Wildlife Sanctuary (KWLS), Western Ghats, India (Table 1; Supplementary Table S1; Supplementary Fig. S1), (2) assess the contribution of each component to the total ecosystem-level C stock, and (3) determine the correlations between C stocks and diversity, stand structural attributes, and environmental factors.

**Table 1:** Study site characteristics, mean ecosystem-level aboveground and belowground biomass (Mg/ha) of different components at seven selected forest sites of KWLS, Western Ghats, India.

Parameter	Tropical dry deciduous		Tropical semi-evergreen		Tropical evergreen			Mean total	*P*-value
	TDD I	TDD II	TSE I	TSE II	TEF I	TEF II	TEF III		
Latitude	8.449	8.524	8.539	8.524	8.536	8.540	8.541	–	–
Longitude	77.320	77.291	77.313	77.289	77.321	77.318	77.311	–	–
Elevation (m MSL)	115–229	317–392	532–630	306–431	521–677	508–655	477–544	–	–
Tree species richness (No./4,000 m^2^)	55	24	45	67	56	44	76	151	0.0001
Tree density (No./ha)	370	448	525	775	900	863	850	675.9	0.0001
Tree basal area (m^2^/ha)	24.2	30.8	35.1	45.7	49.05	64.6	75.3	46.4	0.0001
Mean DBH of adults (cm)	24.9 ± 1.21	26.2 ± 1.03	25.9 ± 0.92	23.3 ± 0.83	23 ± 0.68	26.1 ± 0.91	28.1 ± 1.02	25.4 ± 0.9	0.0001
**Aboveground biomass (Mg/ha)**									
Tree juveniles (< 10 cm)	3.96 ± 0.7^ab^	1.23 ± 0.2^c^	4.66 ± 0.59^ab^	2.20 ± 0.3^bc^	5.82 ± 0.6^a^	5.35 ± 0.6^a^	6.07 ± 0.6^a^	4.18	0.0001
Tree adults (≥ 10 cm)	160.8 ± 16^d^	216.7 ± 18^cd^	364.5 ± 44^bc^	282 ± 27.8^cd^	502.1 ± 46^b^	708.2 ± 51.8^a^	868.2 ± 76.6^a^	443.2	0.0001
Woody climbers	7.4 ± 3.5^ab^	0.03 ± 0.01^b^	0.001 ± 0.0^b^	2.5 ± 0.9^ab^	10.2 ± 3.6^a^	5.5 ± 2.4^ab^	2.8 ± 2^ab^	4.06	0.015
Bamboo and palms	0.2 ± 0.1^a^	–	–	–	0.01 ± 0.01^a^	0.02 ± 0.02^a^	–	0.03	0.080
Understorey	0.33 ± 0.05^c^	36.9 ± 1.63^a^	3.16 ± 0.4^b^	–	–	–	–	5.77	0.0001
Litter	2.5 ± 0.17^c^	2.1 ± 0.13^c^	5.8 ± 0.43^b^	7.1 ± 0.40^ab^	5.8 ± 0.54^b^	7.7 ± 0.22^a^	6.9 ± 0.43^ab^	5.44	0.00
Deadwood	27.6 ± 9.2^c^	7.3 ± 3.9^c^	53 ± 23.2^b^	36.1 ± 9.1^a^	40.8 ± 18.9^b^	58.1 ± 16.9^a^	15.4 ± 3.6^a^	34.03	0.11
**Belowground biomass (Mg/ha)**									
Tree juveniles (< 10 cm)	1.03 ± 0.1^ab^	0.32 ± 0.06^c^	1.21 ± 0.1^ab^	0.57 ± 0.08^bc^	1.51 ± 0.1^a^	1.39 ± 0.1^a^	1.58 ± 0.1^a^	1.09	0.0001
Tree adults (≥ 10 cm)	25.7 ± 2.2^e^	34.6 ± 2.5^de^	55.5 ± 6.4^cd^	45.6 ± 4.3^de^	76.4 ± 7.2^bc^	101.8 ± 6.3^ab^	121.6 ± 9.8^a^	65.9	0.0001
Woody climbers	1.93 ± 0.9^ab^	0.01 ± 0.0^b^	0.0002 ± 0^b^	0.66 ± 0.2^ab^	2.66 ± 0.9^a^	1.43 ± 0.6^ab^	0.73 ± 0.5^ab^	1.06	0.015
Bamboo and palms	0.05 ± 0.03^a^	–	–	–	0.0^a^	0.01 ± 0.0^a^	–	0.01	0.080
Understorey	0.09 ± 0.01^c^	9.6 ± 0.42^a^	0.82 ± 0.11^b^	–	–	–	–	1.50	0.0001

Mean value ± standard error. Mean values within a row followed by different lower case letters are significantly different.

## Results

### Biomass

The mean total live tree biomass ranged from 191.5 (TDD I) to 997.5 (TEF III) Mg/ha across the seven selected forest sites. The mean aboveground biomass (AGB) of tree juveniles ranged 1.23 (TDD II) – 6.07 (TEF III) Mg/ha, while that of adult trees varied between 160.8 (TDD I) and 868.2 (TEF III) Mg/ha (Table 1). The AGB values varied significantly (*P* < 0.0001) among different forest types and also among different sites of the same forest type. Since belowground biomass was estimated from the AGB, the former showed the same trend as the latter.

### Carbon stocks

The tree (above- and belowground) C stock (juveniles + adults) ranged from 85.3 (TDD I) to 444.2 (TEF III) Mg C/ha (mean 229.08 Mg C/ha). Among all forest types, the tropical evergreen forest type had the highest C stocks compared to the dry deciduous and semi-evergreen forest types. In the case of the tropical dry deciduous forest type, TDD II (112.6 Mg C/ha) had higher tree C stock (juveniles + adults) than TDD I (85.3 Mg C/ha), while TSE I (189.7 Mg C/ha) was greater than TSE II (147.1 Mg C/ha) in the tropical semi-evergreen forest type. Among the tropical evergreen forest sites, TEF I (260.8 Mg C/ha) had the lowest, TEF II (363.7 Mg C/ha) had moderate and TEF III (444.2 Mg C/ha) had the highest tree C stocks. Overall, around 86.8% of the total woody biomass C stock is constituted by aboveground C and the rest by belowground C.

Overall, the total vegetation C stocks varied markedly from 89.8 (TDD I) to 445.7 Mg C/ha (TEF III), with the mean value of 234.3 Mg C/ha (Table 2). The major C stock contributors were trees (85.3–444.2 Mg C/ha), followed by understorey (0.17–19.07 Mg C/ha), woody climbers (0.003–5.74 Mg C/ha), bamboo and palms (0.005–0.11 Mg C/ha). The total vegetation C stocks were in the following order: TEF III > TEF II > TEF I > TSE I > TSE II > TDD II > TDD I. *Terminalia paniculata* (40.2% in TDD I, 32.6% in TDD II and 20.4% in TSE II) and *Hopea parviflora* (77.9% in TEF I, 62.2% in TEF II and 61.7% in TEF III) showed the highest C storage in three forest sites, whereas *Pterocarpus marsupium* was the topmost C accumulator in TSE I (38.4%). The percentage contribution of top five tree species to the total vegetation C stock varied amongst seven sites: 66.2% in TDD I, 77.4% in TDD II, 84% in TSE I, 67% in TSE II, 91.2% in TEF I, 80% in TEF II and 75.3% in TEF III. The C storage in litter ranged from 0.9 (TDD II) to 3.5 Mg C/ha (TEF II) with an average of 2.4 Mg C/ha (Table 2). The mean deadwood C stock across all the sites was 15.2 Mg C/ha and it ranged from 3.3 (TDD II) to 25.9 (TEF II) Mg C/ha. The total detritus C stock ranged between 4.3 (TDD II) and 29.4 (TEF II) Mg C/ha (mean 17.6 Mg C/ha).

**Table 2:** Mean ecosystem-level C stocks (Mg C/ha) of different components at seven selected forest sites of KWLS, Western Ghats, India.

Component	Tropical dry deciduous		Tropical semi-evergreen		Tropical evergreen			Mean total	*P*-value
	TDD I	TDD II	TSE I	TSE II	TEF I	TEF II	TEF III		
Tree juveniles (< 10 cm)	2.22 ± 0.4^ab^	0.69 ± 0.1^c^	2.62 ± 0.3^ab^	1.24 ± 0.1^bc^	3.2 ± 0.3^a^	3 ± 0.3^a^	3.4 ± 0.3^a^	2.3	0.0001
Tree adults (≥ 10 cm)	83.08 ± 8^d^	111.9 ± 9^cd^	187.1 ± 22.6^bc^	145.9 ± 14^cd^	257.6 ± 23^b^	360.7 ± 25.9^a^	440.8 ± 38.5^a^	226.7	0.0001
Woody climbers	4.17 ± 9.9^ab^	0.01 ± 0.01^b^	0.003 ± 0.0^b^	1.41 ± 0.4^ab^	5.74 ± 1.9^a^	3.08 ± 1.3^ab^	1.58 ± 1.1^ab^	2.3	0.010
Bamboo and palms	0.11 ± 0.07^a^	–	–	–	0.01 ± 0.01^a^	0.01 ± 0.01^a^	–	0.02	0.080
Understorey	0.17 ± 0.03^c^	19.07 ± 0.8^a^	1.63 ± 0.21^b^	–	–	–	–	3.0	0.0001
Litter	1.14 ± 0.08^c^	0.96 ± 0.06^c^	2.62 ± 0.19^b^	3.19 ± 0.18^ab^	2.63 ± 0.24^b^	3.49 ± 0.10^a^	3.12 ± 0.19^ab^	2.4	0.0001
Deadwood	12.3 ± 4.1^c^	3.3 ± 1.7^c^	23.6 ± 10.3^b^	16.1 ± 4.1^a^	18.2 ± 8.4^b^	25.9 ± 7.5^a^	6.8 ± 1.6^a^	15.2	0.0001

Mean value ± standard error. Mean values within a row followed by different lower case letters are significantly different.

### Soil organic carbon pool

Soil organic C varied significantly (*P* < 0.0001) among study sites. The mean SOC was 84.9 Mg C/ha. The site TEF III (58 Mg C/ha) had the lowest SOC, while the site TDD I (123.6 Mg C/ha) had the highest SOC at 0–30 cm soil depth (Table 3). The SOC stocks decreased with increase in soil depth.

**Table 3:** Total ecosystem-level biomass and C stocks of different components at selected forest sites of KWLS, Western Ghats, India.

Parameter	Tropical dry deciduous		Tropical semi-evergreen		Tropical evergreen			Mean total	*P*-value
	TDD I	TDD II	TSE I	TSE II	TEF I	TEF II	TEF III		
**Total biomass (Mg/ha)**									
Live tree biomass	191.5 ± 18.3^d^	252.9 ± 20.6^cd^	425.9 ± 50.8^bc^	330.4 ± 32.1^cd^	585.8 ± 53.1^b^	816.8 ± 57.7^a^	997.5 ± 86.2^a^	514.4	0.0001
Woody biomass	201.1 ± 20.3^d^	253.0 ± 20.6^cd^	425.9 ± 50.8^bc^	333.6 ± 32.4^cd^	598.7 ± 55.2^b^	823.8 ± 59.2^a^	1,001 ± 85.9^a^	519.6	0.0001
Live vegetation biomass	201.5 ± 20.2^d^	299.5 ± 20^cd^	429.9 ± 42.6^bc^	333.6 ± 32^cd^	598.7 ± 55^b^	823.8 ± 59^a^	1,001 ± 85.9^a^	526.8	0.0001
Detritus biomass	30.2 ± 9.1^ab^	9.4 ± 3.9^b^	58.8 ± 23^b^	43.1 ± 9^ab^	46.6 ± 18.7^a^	65.8 ± 16.8^ab^	22.3 ± 3.7^ab^	39.4	0.070
Total ecosystem biomass	231.7 ± 27.3^d^	308.9 ± 22^cd^	488.7 ± 57.7^bc^	376.7 ± 35^cd^	645.4 ± 59^b^	889.6 ± 65.1^a^	1,023.3 ± 85.5^a^	566.3	0.0001
**Total carbon (Mg C/ha)**									
Live tree carbon	85.3 ± 8.1^d^	112.6 ± 9^cd^	189.7 ± 22.6^bc^	147.1 ± 14^cd^	260.9 ± 23^b^	363.7 ± 25.7^a^	444.2 ± 38.3^a^	229.08	0.0001
Woody biomass carbon	89.6 ± 9.0^d^	112.6 ± 9.1^cd^	189.7 ± 22.6^bc^	148.6 ± 14.4^cd^	266.7 ± 24.5^b^	366.8 ± 26.3^a^	445.7 ± 38.2^a^	231.3	0.0001
Live vegetation carbon	89.8 ± 9.0^d^	131.7 ± 9.1^cd^	191.3 ± 22.6^bc^	148.6 ± 14.4^cd^	266.7 ± 24.5^b^	366.8 ± 26.3^a^	445.7 ± 38.2^a^	234.3	0.0001
Detritus carbon	13.4 ± 4.0^c^	4.3 ± 1.7^c^	26.2 ± 10.2^b^	19.3 ± 4.1^a^	20.8 ± 8.3^b^	29.4 ± 7.5^a^	9.9 ± 1.6^a^	17.6	0.060
SOC (0–30 cm)	123.6 ± 7.6^a^	107.6 ± 3.1^a^	78.3 ± 2.6^b^	59.6 ± 2.6^c^	82.6 ± 2.2^b^	84.7 ± 3.3^b^	58 ± 4.3^c^	84.9	0.0001
Total ecosystem carbon	226.8 ± 12.7^c^	243.5 ± 10.9^c^	295.9 ± 26.2^bc^	227.4 ± 15.5^c^	370.0 ± 26.9^b^	480.9 ± 29.4^a^	513.7 ± 37.2^a^	336.8	0.0001

Mean value ± standard error. Mean values within a row followed by different lower case letters are significantly different.

### Total ecosystem-level C stock

**Figure 1:**
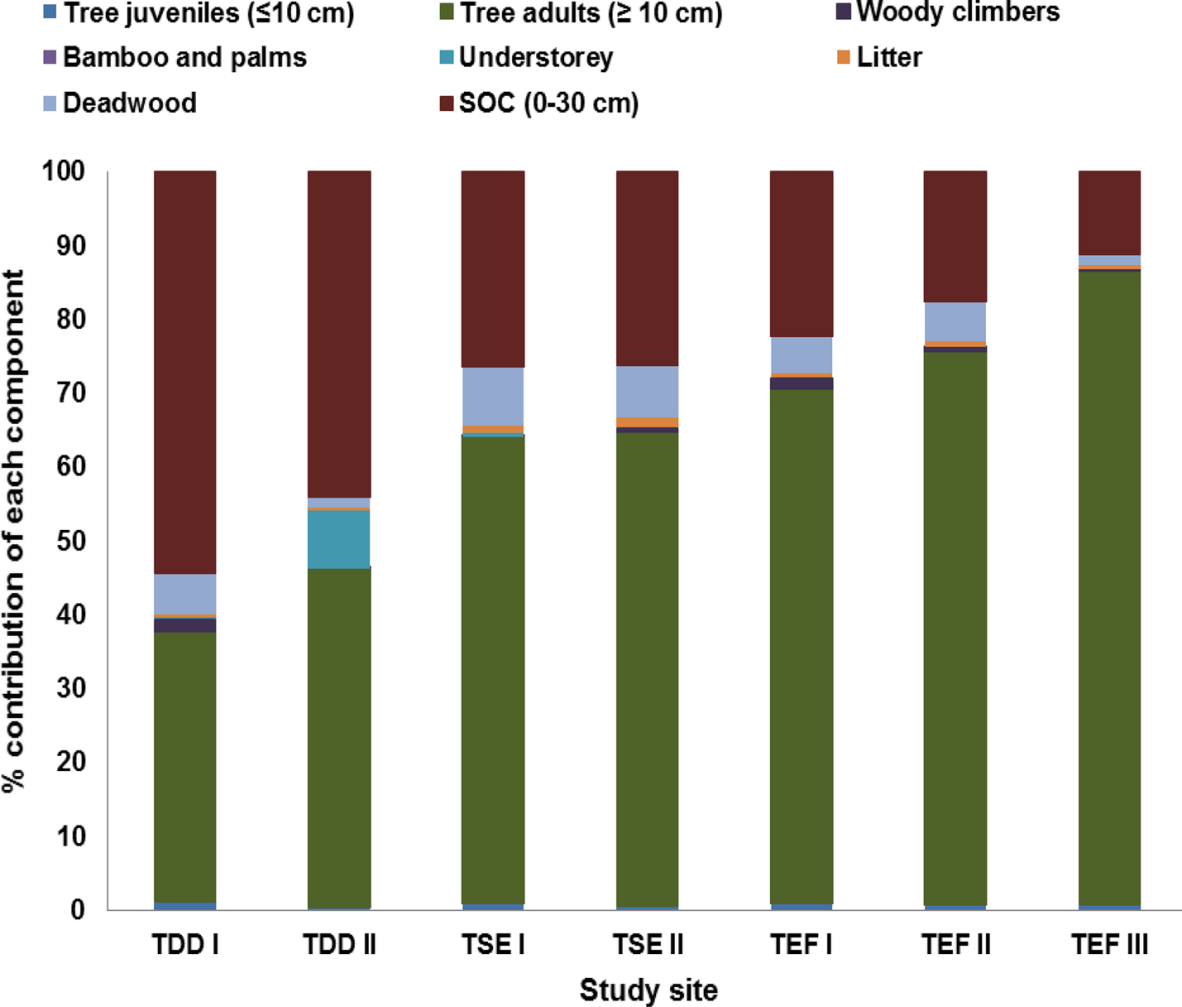
Percent contribution of different ecosystem components to the total ecosystem C at seven selected forest sites in KWLS, Western Ghats, India.

The C stock of total woody vegetation, understorey, litter, deadwood and SOC varied significantly (*P* < 0.0001) among study sites (Table 3). The highest total ecosystem C stock was found in TEF III (513.7 Mg C/ha) and the lowest was observed in TDD I (226.8 Mg C/ha) with the mean total ecosystem C stock of 336.8 Mg C/ha. Overall, TEF (78%), TDD (47%) and TSE (9%) forest types had the highest vegetation, SOC and detritus C stocks respectively. Each forest type showed different C allocation patterns among different ecosystem components. The percentage contributions of tree juveniles, adults, woody climbers, bamboo and palms, understorey, detritus and SOC to the total ecosystem C stock were 0.7, 62.9, 0.7, 0.01, 1.2, 5.5 and 29% respectively (Fig. 1).

### Relationships between C stocks and ecosystem parameters

**Figure 2:**
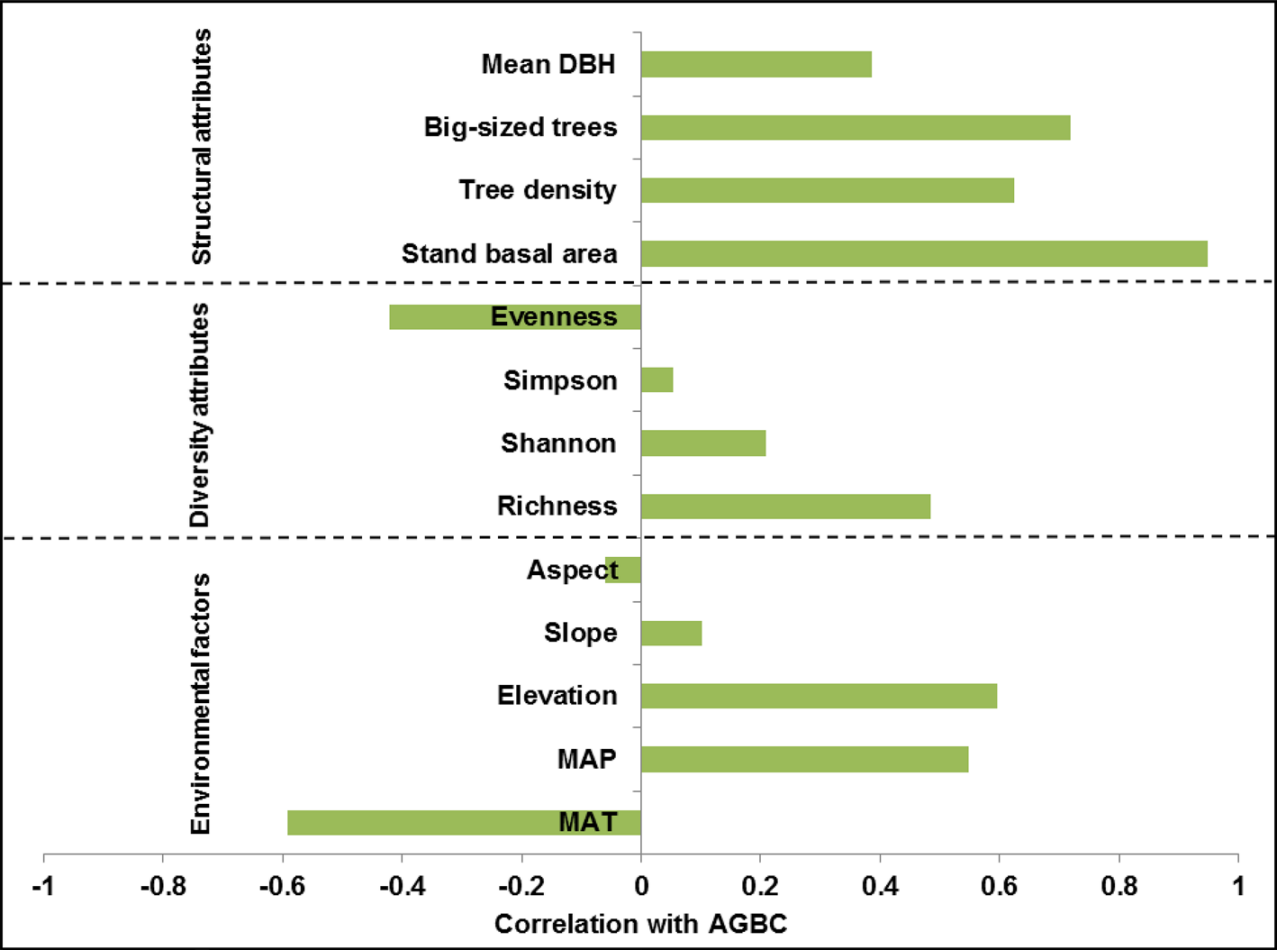
Correlation between aboveground biomass C (AGBC) and environmental factors{topographic variables: elevation, slope, aspect; climatic variables: mean annual temperature (MAT), mean annual precipitation (MAP)}, diversity attributes{species richness (richness), species evenness (evenness), Shannon’s diversity index (Shannon), Simpson’s dominance index (Simpson)} and structural attributes {stand basal area, tree density, big-sized trees (≥ 60 cm DBH), mean tree DBH}.

Among diversity attributes, AGBC had significant positive correlation with tree species richness (r = 0.484; *P* < 0.0001) and negative correlation with species evenness (r = − 0.421; *P* < 0.0001; Table 4; Fig. 2; Supplementary Fig. S2a,b). Among structural attributes, significant positive relationships of AGBC were observed with stand basal area (r = 0.948; *P* < 0.0001), big-sized trees (r = 0.718; *P* < 0.0001), tree density (r = 0.624; *P* < 0.0001) and mean tree DBH (r = 0.386; *P* < 0.001). Among environmental factors, significant positive correlations of AGBC were observed with elevation (r = 0.597; *P* < 0.0001) and MAP (r = 0.549; *P* < 0.0001), and a significant negative correlation with MAT (r = − 0.592; *P* < 0.0001). The relationships of AGBC with slope, aspect, Shannon’s diversity index and Simpson’s dominance index were non-significant.

**Table 4:** Correlations (r-values) between C stocks of different components and predictor variables in the selected forest sites of KWLS, Western Ghats, India (*df* degrees of freedom).

Predictor variable	r-value					
	Juvenile carbon (df = 70)	Adult carbon (df = 70)	Tree carbon (df = 70)	Woody climber carbon (df = 51)	Bamboo and palm carbon (df = 8)	Total woody carbon (df = 70)
No. of juveniles	0.999****	0.431****	0.439****	– 0.078	– 0.155	0.436****
Juvenile basal area	0.939****	0.399***	0.406****	– 0.043	– 0.421	0.405***
No. of adults	0.339**	0.625****	0.626****	0.220	– 0.059	0.631****
Adult basal area	0.321**	0.948****	0.948****	0.098	– 0.382	0.948****
No. of trees	0.918****	0.599****	0.605****	0.034	– 0.152	0.605****
Tree basal area	0.370**	0.954****	0.953****	0.094	– 0.394	0.954****
No. of woody climbers	0.105	– 0.188	– 0.187	0.568****	– 0.276	– 0.167
Woody climber basal area	– 0.055	0.008	0.008	0.961****	– 0.243	0.040
No. of bamboo and palms	– 0.132	– 0.398	– 0.399	0.311	0.709*	– 0.391
Bamboo and palm basal area	– 0.152	– 0.393	– 0.394	– 0.322	0.906**	– 0.412
No. of juvenile species	0.802****	0.572****	0.577****	– 0.046	0.350	0.574****
No. of adult species	0.307**	0.478****	0.479****	– 0.042	– 0.078	0.478****
No. of tree species	0.622****	0.619****	0.623****	– 0.097	0.191	0.619****
No. of woody climber species	– 0.008	– 0.210	– 0.210	– 0.065	– 0.024	– 0.211
No. of bamboo and palm species	– 0.436	– 0.777*	– 0.779*	0.207	0.367	– 0.780*
Litter carbon	0.459**	0.637****	0.639****	0.055	– 0.185	0.638****

r-values with asterisk represent a significant relationship between the variables (*P < 0.05; **P < 0.01; ***P < 0.001; ****P < 0.0001; Other r-values exhibit relationships which are non-significant).

**Figure 3:**
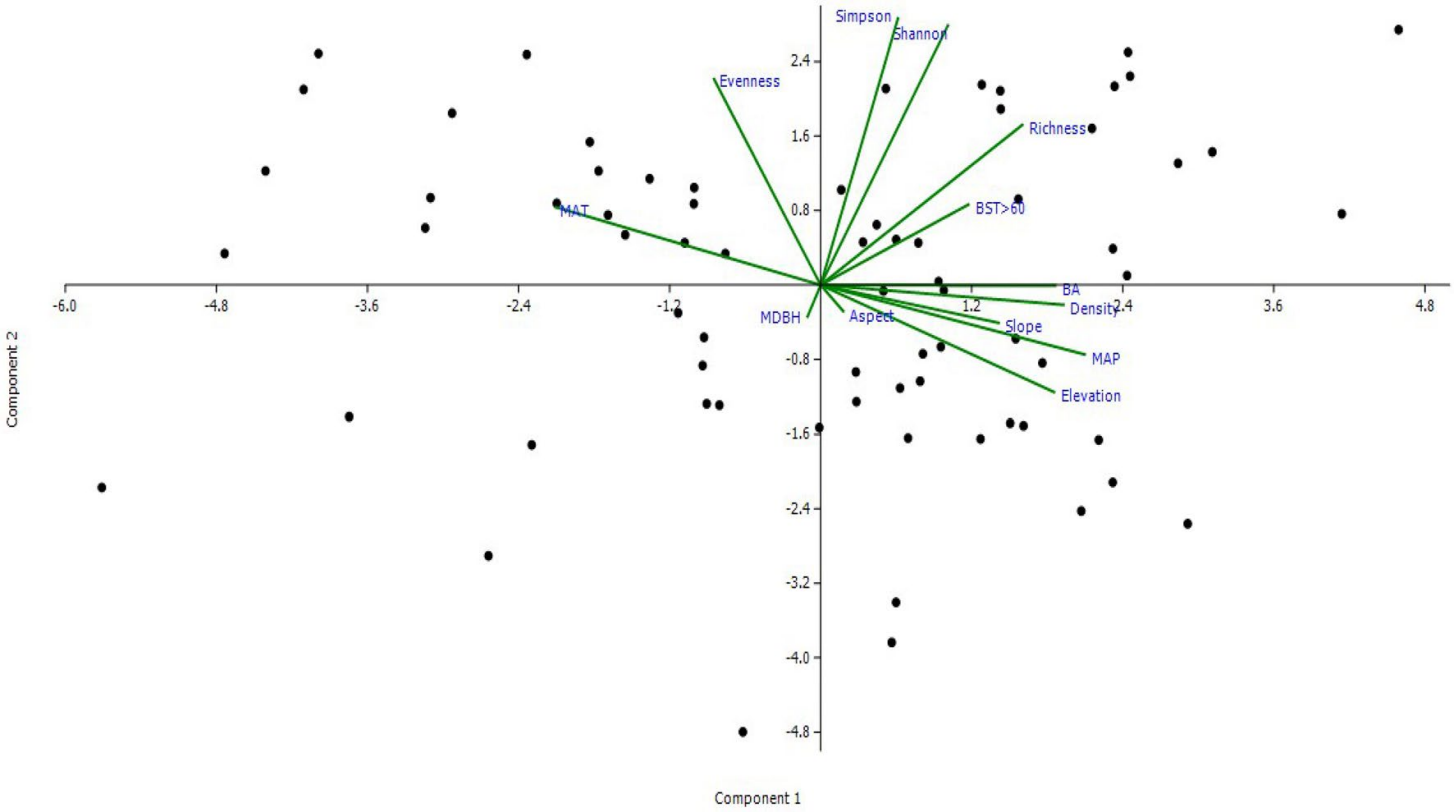
Principal components analysis of environmental factors {topographic variables: elevation, slope, aspect; climatic variables: mean annual temperature (MAT), mean annual precipitation (MAP)}, diversity attributes {species richness (richness), species evenness (evenness), Shannon’s diversity index (Shannon), Simpson’s dominance index (Simpson)} and structural attributes {tree basal area (BA), tree density (density), big-sized trees ≥ 60 cm DBH (BST > 60), mean tree DBH (MDBH)} against forest aboveground biomass C at plot-level. Each dot represents a plot.

The Principal Components Analysis (PCA) was performed to assess the relationships among diversity, structural attributes and environmental factors with AGBC at plot-level (Fig. 3). Eigen values of the dominant axis were 4.84, 2.80, 2.12, 1.09, 0.88 and 0.54 respectively and the corresponding percentage variances were 37.2, 21.5, 16.3, 8.4, 6.7 and 4.1. These values reflect on variations in stand basal area, big-sized trees, tree density, elevation, MAP and tree species richness, which together accounted for 94.3% variation in AGBC revealing that AGBC is influenced by structural attributes, environmental factors and diversity attributes.

## Discussion

Biomass and C stocks are important quantitative parameters in forest ecology. The differences in assessment protocols including plot shapes and sizes, and tree size thresholds often hinder the comparability of results between studies (Supplementary Table S2). On a continental scale, the mean AGB of adult trees (443.2 ± 99.6 Mg/ha) is comparable to the corresponding mean values of tropical forests of Africa (429 Mg/ha)^38^ and Asia (457.1 Mg/ha)^39^ and greater than that of Amazonia (341 Mg/ha)^38,40^. At the country-level scale, the observed mean value is 37.5% higher than that estimated by Haripriya^41^ for Indian forests (67.4 Mg/ha). The results were also compared with studies with small plot size (< 0.25 ha) to minimize the scale-dependent effects while making comparisons^19^. The observed mean value was higher than the values reported from pine forests (101.3–314.4 Mg/ha)^42^, dry semi-evergreen forests (191.5 Mg/ha)^43^ and a miombo woodland (34.5–65.1 Mg/ha)^44^ in China but lower than a dry forest (720.7 Mg/ha)^45^ in Ethiopia.

**Figure 4:**
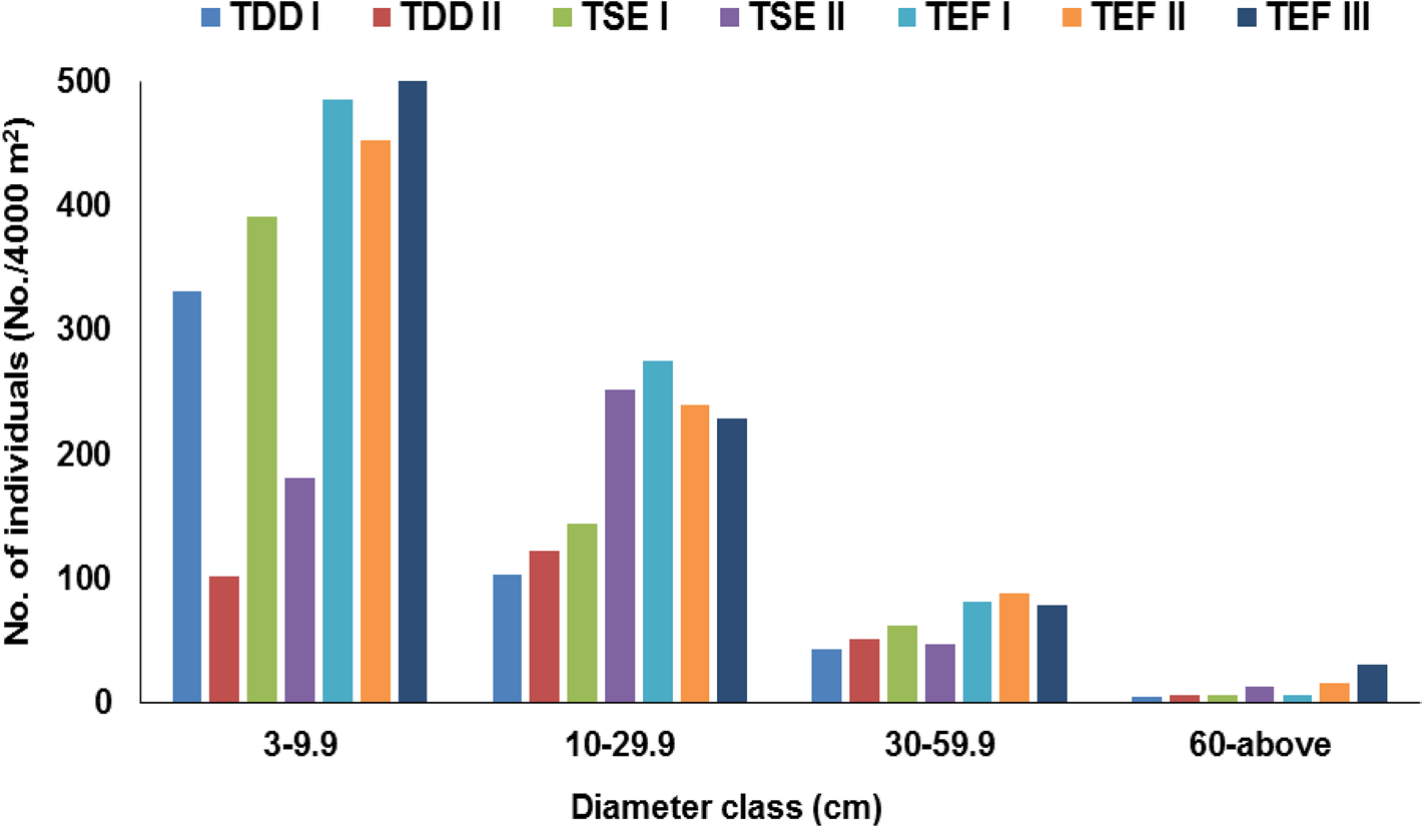
Diameter class distribution of tree density at seven selected forest sites in KWLS, Western Ghats, India.

The values of ecosystem C storage includes vegetation C, detritus C and soil C storage^46^. The above- and belowground C stocks of juveniles and adults varied greatly among different forest types and also within sites of the same forest type. Overall, the tree C stock among forest types was in the order dry deciduous < semi-evergreen < evergreen. The mean tree C stock of the tropical dry deciduous forest type was 98.9 Mg C/ha. The prevalence of hot and dry summers in the dry deciduous sites (MAT: 26.8 °C and 25.7 °C for TDD I and TDD II respectively, 24.3–25.3 °C for other sites; minimum temperatures: 21.6 °C and 20.5 °C for TDD I and TDD II respectively, 19–20.1 °C for other sites; and maximum temperatures: 32.3 °C and 31.1 °C for TDD I and TDD II respectively, 29.8–30.8 °C for other sites)^47^ may have resulted in the suppression of tree growth^48^ leading to a decreased C storage in these sites. The site TDD II which had greater basal area and higher number of tree adults had higher C stock (111.9 Mg C/ha) as compared to TDD I (83.08 Mg C/ha) which had lower basal area and lesser number of tree adults (Fig. 4). This could be attributable to anthropogenic disturbances in TDD I with easier accessibility than TDD II with restricted accessibility (Supplementary Table S1). Thus, variation in stem density and basal area led to considerable variation in tree C storage among the two dry deciduous sites. Our results are in agreement with the findings of Solomon et al*.*^49^, McNicol et al*.*^50^, Dimobe et al*.*^51^ and Raha et al*.*^52^ from other tropical dry forests elsewhere. Therefore, from the conservation standpoint, these tropical dry deciduous sites which hold substantial C stocks need to be protected from disturbances and degradation to enhance their C storage. As tropical dry forest area is shrinking nowadays due to deforestation, disturbances, conversion to plantations, etc., accurate accounting of C stocks in these forests are important for climate change mitigation and forest conservation wisely and sustainably. Hence, these results would be helpful in understanding the role of dry forests in regional and global C cycling, and can be used in forest management activities to boost C sequestration and have great implications for conservation planning.

The semi-evergreen forest type accrued moderate amounts of tree C stock (mean: 168.4 Mg C/ha) as these sites neither suffer disturbances like the dry deciduous forest type nor are bestowed with proximity to perennial rivulets/streams like the evergreen forest type. The differences in the adult tree C stocks of two sites (TSE I: 187.1 Mg C/ha, TSE II: 145.9 Mg C/ha) reflect the differences in species composition, tree density and basal area. Although the tree density and basal area are more in TSE II than TSE I, the mean DBH value of TSE I is greater than that of TSE II. This is attributable to higher number of mid-sized stems in TSE I (61 stems/4,000 m^2^) than TSE II (46 stems/4,000 m^2^), which in turn resulted in greater C stocks in TSE I (Fig. 4).

The tropical evergreen forest type had the highest mean tree C stock (356.2 Mg C/ha). This is due to the presence of large trees, strongly influenced by availability of water from perennial streams. The water availability influences the stand structure and basal area of forests^48^. Among evergreen forest sites, TEF I (257.6 Mg C/ha) had the least adult tree C stock. This is due to the presence of high number of small diameter-class (10–30 cm DBH) trees compared to other evergreen sites, which indicates that this site may stock more C in the future (Fig. 4). In contrast, the highest adult tree C stock was found in TEF III (440.8 Mg C/ha), which could be attributable to the presence of highest number of big-sized trees (60–100 cm DBH). Lutz et al*.*^53^ reported that big-sized trees (≥ 60 cm DBH) account for 41% of AGB (and therefore C) on a global level. In this study, big-sized trees (≥ 60 cm DBH) contributed 16.2% (TDD I) to 47.4% (TEF III) of the tree C. In general, big-sized trees consist of large, well-lit crowns that contribute to high primary productivity of forests^8^ and therefore store more C.

The C stocks in woody climbers ranged from 0.003 to 5.7 Mg C/ha, which is similar to values in pantropical forests (5.58 Mg C/ha)^54^. However, TEF I had higher C stocks (5.7 Mg C/ha) than TDD I (4.2 Mg C/ha), although the latter had more diversity and density (TEF I—3 species/4,000 m^2^, 198 stems/ha; TDD I—10 species/4,000 m^2^, 358 stems/ha). The low woody climber density in the evergreen sites could be due to their considerable distance from human settlements, restricted entry and higher protection than the other sites, and therefore trees in TEFs I–III remain undisturbed, with less overgrowth of woody climbers. Woody climber density was negatively associated with the C stocks of trees (≥ 10 cm) in tropical forests^54^. Similar results were observed in the present study, where TDD I, which had the highest woody climber density had the lowest adult tree C stocks.

The understorey C stocks ranged from 0.17 (TDD I) to 19.07 (TDD II) Mg C/ha. The several fold high C stocks in the understorey in TDD II is due to the dominance of the tall and clumped grass species (*Themeda cymbaria*). This site was previously subjected to annual ground fires, which may have favoured the growth of this tall grass species (Supplementary Table S1). Furthermore, the site TDD II had a sparse distribution of trees leading to unhindered influx of sunlight that in turn led to the high growth of the grass species. The mean total live vegetation C stocks ranged from 89.8 (TDD I) to 445.7 Mg C/ha (TEF III) with the mean value of 234.3 Mg C/ha, which are higher than the estimated values in Asian tropical forests (144 Mg C/ha)^55^. The mean value in our study is within the range of 50–360 Mg C/ha, similar to tropical forests of Southeast Asia^56^, and overall could be considered as highly C-dense forests.

Different vegetation components contributed varying proportions to the total vegetation C stocks in different sites. Overall, trees contributed 96.5% (adult trees—95.3%, juvenile trees—1.2%) to the total vegetation C stock, followed by understorey (2.2%), woody climbers (1.3%), and bamboo and palms (0.02%). It is well-known that trees, especially big-sized trees often contain high biomass and therefore function as C accumulators^57^. The contribution of adult trees is > 96% in the semi-evergreen and the evergreen forest sites, but not in the tropical dry deciduous sites (TDD I—92%, TDD II—84%). In TDD II, the contribution of adult trees has been substantially reduced due to the prevalence of monodominant understorey (*Themeda cymbaria*). The contribution of juvenile trees was higher than the understorey in all the sites, except TDD II and ranged between 0.5 and 2.4%. The high growth of grass in TDD II suppressed the growth of juvenile trees, and the establishment of latter is further hindered due to occasional ground fires. This led to contribution of juvenile trees to total vegetation C to be low with only 0.5% in this site. However, despite the fact that overall contribution of juvenile trees to C stocks is low with ~ 1%, they still form an important component of the C pool as they may serve as potential C reserves in future. The understorey C stock contributed as much as 14.5% in TDD II and this proves that the understorey vegetation has a great potential for C storage not only within itself, but also by influencing other components as well. The contribution of woody climbers to C stock was highest in TDD I (4.6%), while woody climbers of TEF I and II also contributed substantially with 2.2 and 0.8% respectively. This is possibly due to their higher mean DBH (> 5 cm) in the evergreen forest sites. The high contribution of woody climbers to C stock in TDD I could be attributable to anthropogenic pressures as this site is located along human settlements. Woody climber density and C is known to increase with disturbance at edges^58^. The bamboos are not efficient C sinks as they may soon release the stored C as individual culms are short-lived with 7–10 years^59^ of lifespan. The contribution of bamboos to C stocks is very low (0.002–0.09%) and it is absent in most sites. The contribution of palms was only 0.003–0.03%, possibly due to their unique architecture, anatomy with no secondary growth^60^. Although the contribution of bamboo and palms to C stock is negligible in this study, these serve as important components in the overall C stock of the forest vegetation.

Although C stock in litter accounts for about 5% of C storage in forest ecosystems^2,46^, our present knowledge on C stocks is limited^61^. In this study, the C in litter ranged 0.9–3.5 Mg C/ha, with a mean of 2.4 Mg C/ha. The observed mean is close to the values of tropical forests reported by Brown and Lugo^62^ (2.6–3.8 Mg C/ha). The deadwood is also an important C reservoir and its decomposition could lead to C release to the atmosphere and its magnitude varies with forest type^63,64^. The C stocks in deadwood varied between 3.3 and 25.9 Mg C/ha, with a mean of 15.2 Mg C/ha. The mean C stocks in deadwood is lower than that reported by Tran and Dargusch^65^, but higher than other studies^56,66^. The deadwood was the third largest C pool after the C pools in vegetation and soil in this study. Altogether, the C stock in detritus averaged 17.6 Mg C/ha and serves as an important pool linking vegetation and soil C pools^46,67^.

Soil organic carbon is an important component of the C pool and its storage potential varies with the forest type. In the present study, the SOC stock ranged from 58 to 123.6 Mg C/ha, with a mean of 84.9 Mg C/ha. The observed mean is within the range reported for soil C in the tropical forests of Asia (50–120 Mg C/ha)^68^ and closer to the range of 104.9–165.2 Mg C/ha reported from Chinnar Wildlife Sanctuary^69^. The SOC stocks were highest in the topmost layer (0–10 cm) and it decreased with increase in soil depth. This could be because the surface soil usually has high humus content^70^. Also, the decrease in SOC with depth could be attributed to soil compaction and slower cycling of SOC pools^71^. The SOC stocks were found to be high in the tropical dry deciduous sites and less in the semi-evergreen and evergreen sites. Such a variation in SOC stocks could be attributable to the variability in vegetation composition, quality and quantity of litter input, soil bulk density, soil parent substrate and microclimate^71,72^.

An accurate estimation of ecosystem C stocks and their distribution among different components is critical for understanding the global C cycle^66^. In the present study, our results showed that the tropical forest ecosystem C stocks ranged from 226.8 to 513.7 Mg C/ha, with a mean of 336.8 Mg C/ha. The observed mean ecosystem C stock is higher than the tropical forests of China (214 and 303 Mg C/ha)^56,66^ and comparable with the C stock of a primary forest in Singapore (337 Mg C/ha)^73^. However, the results are lower than other studies with plot sizes of < 0.25 ha^45,74^. Overall, TEF (78%), TDD (47%) and TSE (9%) forest types had the highest vegetation, SOC and detritus C stocks respectively. Among tropical forest types, C stocks in rainforests have been extensively, while the dry forests remained poorly studied^75,76^. The present study showed that tropical dry deciduous forest sites also serve as major C sinks by holding substantial amounts of C stock in soil. The surface soils of tropical dry forest is rich in soil microbial activity, soil nutrients and has considerable amount of C^76^. Thus, the tropical dry forests, one of the mostly threatened ecosystems^77^ should not be ignored in C accounting just merely based on AGB and it should be prioritized for conservation and C stocking purposes.

In this study, the allocation patterns of C varied greatly among the different ecosystem components in different study sites (Fig. 1). On average, trees, woody climbers, bamboo and palms, understorey, forest floor litter, deadwood and soil accounted for 63.6, 0.7, 0.01, 1.2, 0.7, 4.8 and 29% of the total ecosystem C respectively. Overall, the ecosystem C pool among the different ecosystem components was in the order vegetation (65.5%) > soil (29%) > detritus (5.5%). Similar results were observed in other tropical forests as well^45,56,66,74^. Soil was the major C pool in the dry deciduous site TDD I (54%), while the soil of TDD II also held substantial levels of C (44%). This could be due to deciduousness of trees, which therefore lead to high organic matter inputs in these sites. On the other hand, the semi-evergreen and evergreen forest sites had maximum C stocks in the vegetation (≥ 64%), due to presence of relatively big-sized trees. The highest contribution of vegetation to the total ecosystem C was observed in the tropical evergreen site, TEF III (86.8%) due to extensive presence of big-sized trees. In the present study, the detritus C pool constituted only 1.7–8.9% (average 5.5%) of the total ecosystem C. However, with the ever-increasing atmospheric CO_2_ levels, any pool with a potential to lock C should be included in C budgets and hence the detritus pool should not be treated as trivial.

Forest ecosystem functioning is determined by environmental factors, diversity and structural attributes^8^. Although correlation does not necessarily mean causation^28^, correlation reveals potential relationships between variables, which needs further studies to establish causal relationships. Among diversity attributes, AGBC was significantly (*P* < 0.0001) and positively correlated with tree species richness and negatively with species evenness (Fig. 2). Tree species richness is known to increase tree size inequality among and within species, and therefore enhances C stocks in a forest, while tree species evenness has a negative effect^28,78^. Significant positive relationships (*P* < 0.001–*P* < 0.0001) were observed between AGBC and all structural attributes studied. High tree densities enhance C stocks due to greater canopy packing leading to more light harvesting and increased wood production^79^. As biomass increases exponentially with tree diameter, high stand basal area and big-sized tree densities increase C storage^8^. At spatial scales of 0.04 ha, as used in the present study, the positive relationship between species richness and C stocks could be attributed to local variation in stem density^19^, large tree sizes^57^ and dominance effects^80^. In the present study, the highest AGBC was observed in TEF III, the site with the highest basal area, greatest mean DBH and highest number of big-sized trees (Table 1; Fig. 4). Among environmental factors, AGBC was significantly (*P* < 0.0001) and positively correlated with elevation and MAP, and negatively with MAT. Elevation influences AGBC by affecting microclimate, which in turn influences stand attributes^78,81^. AGBC is also influenced by MAP as high annual rainfall increases the length of the growing season, enhancing the growth of trees, leading to increased biomass^82^. On the other hand, high MAT often has a limiting effect on stand C stocks^26^. Similar results were observed in other studies as well. Vayreda et al*.*^26^ found that species richness and structural richness variables are better predictors of C accumulation than climatic and local site variables in Western Mediterranean region. Poorter et al*.*^8^ observed that diversity attributes are strongly related to AGB at small spatial scales, whereas structural attributes are related to AGB at all spatial scales. They also concluded that rainfall is a major driver of AGB. Behera et al*.*^27^ reported a strong positive relationship of AGB with species richness and structural attributes in an Indian tropical deciduous forest. Poorter et al*.*^28^ showed that vegetation attributes were more frequently and significantly associated with biomass than environmental conditions. Liu et al*.*^29^ found that tree species richness enhances ecosystem-level C storage in the subtropical forests of China. Amara et al*.*^30^ reported a moderate linear relationship between tree species richness and AGBC in the Guinean savanna landscape, Africa. Li et al*.*^31^ observed that tree C storage was significantly positively related with diversity, structural attributes and precipitation. The PCA analysis also showed that AGBC is positively influenced by MAP, tree density, basal area, elevation and species richness, and negatively affected by MAT and species evenness as major variables having maximum PCA component matrix. Diversity, structural attributes and environmental factors accounted for 6.7, 77.2 and 16% of variance in AGBC.

In the present study, trees emerged as the most important structural component. Trees, in particular big-sized trees, are regarded as long-standing C sinks as they keep accruing C for centuries during their lifespan, and decompose slowly^83^. Woody climbers contributed to only a small fraction of the ecosystem C as they invest very little in biomass^84^. However, woody climbers have the potential to drastically alter tropical forest C storage as they affect host trees by causing stem deformations, branch breakages and increasing the risk of mortality^84^. Thus, although woody climbers can store C for short periods of time, the impacts they impose on the other major C reservoir, such as trees are considerable. Although bamboos and palms represent small fractions of ecosystem C stocks, they are nevertheless important components of tropical forest C storage^60,85^. Thus, overall, big-sized trees form the most significant component of woody vegetation, which in turn comprises a major fraction of ecosystem C stock and play a crucial role in climate change mitigation strategies^86^. The evergreen forest type had the greatest tree species richness, highest number of big-sized trees and hence, highest C stocks. Therefore, the evergreen forest type has a major scope for both biodiversity conservation and C stock enhancement programmes. However, big-sized trees are more susceptible to sapwood cavitation and drought, and any loss of big-sized trees could dramatically alter forest structure and C storage^86,87^. In this context, the dry deciduous forest type, which although had lower species richness, fewer number of big-sized trees and lower C stocks than evergreen forest type gains substantial importance due to its unique biodiversity with cavitation-resistant and drought-tolerant species^88^. Smaller trees constitute a relatively large proportion of diversity, stem density and C stocks in dry forests^8,87^. Therefore, dry forest types should also be prioritized in the implementation of conservation, restoration and C management schemes. Information on the diversity, structure and C of these forest types provide valuable insights for species conservation and forest management.

## Conclusion

In the present study, the C stocks varied significantly within and among the forest types, and varied among various ecosystem components. Overall, the highest percentage of C stocks in vegetation, soil and detritus were found in TEF (78%), TDD (47%) and TSE (9%) forest types respectively. This study revealed that the evergreen forest type which had the highest tree species richness, basal area and highest number of big-sized trees had the highest C stocks as compared to the dry deciduous and semi-evergreen forest types with low species richness, basal area and low number of big-sized trees. The C stocks are influenced by structural attributes, environmental factors and diversity attributes. Continued conservation of biodiversity and maintenance of big-sized trees and stand structure will enhance C storage in the studied forest types. The findings are invaluable for identifying areas that need further attention for conservation and C stock enhancement in action plans, and realize the Convention on Biological Diversity 2020 targets. This study will also be valuable for gaining insights into the C allocation patterns of different components at an ecosystem-level in tropical forests and to recognize their crucial role in C storage. This study emphasizes the inclusion of all the C pools (vegetation, detritus and soil) and their relationships with environmental factors, diversity and structural attributes in ecosystem-level C accounting of tropical forests.

## Materials and methods

### Study area

This study was carried out in Kanyakumari Wildlife Sanctuary (KWLS) in Tamil Nadu (8°03′–8°35′ N and 77°05′–77°36ʹ E), which is a part of Agasthyamalai Biosphere Reserve, located at the southern tip of Western Ghats. It shares its borders with Neyyar Wildlife Sanctuary of Kerala and Kalakkad-Mundanthurai Tiger Reserve of Tamil Nadu. The forests of Kanyakumari district are virgin and about 75 million years old^89^. Kanyakumari has 1,003.5 km^2^ of forest cover, comprising 137.1 km^2^ of very dense forest, 572.6 km^2^ of moderately dense forest and 293.7 km^2^ of open forest^33^. Altogether, the forests of Kanyakumari represent 59.5% of the district’s geographical area^33^. This wildlife sanctuary was declared in 2002 with an original extent of 45,777.57 ha. However, in 2007, its boundaries have been redefined to an area of 40,239.55 ha. The study area experiences a tropical climate and is highly influenced by south-west and north-east monsoon winds, with a rainy season during October and November and a dry season between March and May. The sanctuary receives an average annual precipitation of 1,329 mm. The mean annual maximum and minimum temperatures are 32.3 °C and 19 °C respectively^47^. The entire sanctuary is hilly, steep and rugged, and the elevation ranges from 50 to 1,650 m a.s.l., and the highest point (1829.4 m) is located at the tri-junction of Mahendragiri, Veerapuli and Kalakad Reserve Forests. The soil is mostly red loamy, and at some places sandy or clayey as well. The sanctuary is traversed with many rivers including Manimuttar, Pachiar, Varattar, Kodayar, Masupattiar and Chittar, which run south-westerly. Due to variations in temperature, rainfall, elevation, terrain and edaphic characteristics, different habitat types occur in this sanctuary. However, no comprehensive study has been carried out in major forest types with respect to C stock assessment of all the pools and therefore an ecosystem-level C stock assessment in relation to diversity, stand structural attributes and environmental factors was carried out at KWLS, Western Ghats, India.

Among different forest types that occur in the Western Ghats, tropical deciduous (moist and dry), semi-evergreen and evergreen forest types are the predominant forest types. The dry deciduous forest type covers an area of 7,239.69 km^2^, while the semi-evergreen and evergreen forest types span 8,314.07 km^2^ and 8,250.08 km^2^ respectively^90^. For the present study, three forest types comprising seven study sites (Table 1; Supplementary Table S1; Supplementary Fig. S1 [land use/land cover data obtained from Bhuvan Thematic datasets, https://bhuvan-app1.nrsc.gov.in/thematic/thematic/index.php]) were selected: tropical dry deciduous forest (TDD I and TDD II), tropical semi-evergreen forest (TSE I and TSE II) and tropical evergreen forest (TEF I, TEF II and TEF III). The study sites under each forest type were mainly selected based on differences in distance and elevation among the forest sites. On the whole, the sites are located in distinct forest ranges across different locations (Pechipaarai, Kutriyar, Sangilippaalam and Paayaasappaarai) in KWLS. The dominant tree species in the study sites are *Terminalia paniculata* and *Pterocarpus marsupium* in TDD I, *Terminalia paniculata* and *Dillenia pentagyna* in TDD II, *Pterocarpus marsupium* and *Ficus beddomei* in TSE I, *Aporosa cardiosperma* and *Ixora pavetta* in TSE II, *Hopea parviflora* and *Vitex altissima* in TEF I, *Hopea parviflora* followed by *Aporosa cardiosperma* and *Diospyros affinis* in TEF II, and *Hopea parviflora* and *Diospyros affinis* in TEF III.

The study area encompasses major forest types of Western Ghats and is also very rich in biodiversity^91^, which makes it an ideal region to study biodiversity-C storage relationships and may be applicable to similar forest types in the tropics. Therefore, accurate estimation of C stocks in this region will have great implications for the implementation of REDD+ policies, especially in terms of biodiversity conservation and C storage.

### Experimental design and field methods

A total of 70 (20 m × 20 m) square sample plots were established in the selected forest sites (ten sample plots in each study site) for documentation of phytosociological attributes as well as biomass and C stock assessment of all the pools. These plots were further subdivided into four 10 m × 10 m quadrats. The plants in 10 m × 10 m quadrats were then classified as follows: juvenile trees (individuals with 3–9.9 cm DBH), adult trees (individuals with ≥ 10 cm DBH) and woody climbers (climbing individuals with girth ≥ 2.5 cm at 1.3 m from the rooting point). For multi-stemmed trees, bole girths were measured separately, basal area were calculated and summed. The study period was from December 2015 to May 2017. The woody plants were enumerated during March–May, while the understorey was documented during its peak growing period in November. The aboveground and belowground biomass were estimated using standard forest type-specific and species-specific biomass regression equations using the wood densities from the Global Wood Density Database^92^.

Aboveground biomass (AGB) equations of adult trees

AGB of trees was estimated following (Alvarez et al*.*^93^; Phillips et al*.*^94^)

$$\begin{aligned} {\text{Dry forest }}\left( {\text{TDDs I and II}} \right){:}\;{\text{AGB}} & = {\exp}\left( {{3}.{652} - \left( {{1}.{697} \times {\ln}\left( {{\text{DBH}}} \right)} \right) + \left( {1.169 \times \left( {{\ln}\left( {{\text{DBH}}} \right)} \right)^{{2}} } \right)} \right. \\ & \quad \left. { - \left( {0.{122} \times \left( {{\ln}\left( {{\text{DBH}}} \right)} \right)^{{3}} } \right) + \left( {{1}.{285} \times {\ln}\left( {\text{wood density}} \right)} \right)} \right) \\ \end{aligned}$$

$$\begin{aligned} {\text{Semi-evergreen forest }}\left( {\text{TSEs I and II}} \right){:}\;{\text{AGB}} & = {\exp}\left( {{2}.{4}0{6} - \left( {{1}.{289} \times {\ln}\left( {{\text{DBH}}} \right)} \right) + \left( {{1}.{169}\left( { \times \left( {{\ln}\left( {{\text{DBH}}} \right)} \right)^{{2}} } \right)} \right)} \right. \\ & \quad \left. { - \left( {0.{122} \times \left( {{\ln}\left( {{\text{DBH}}} \right)} \right)^{{3}} } \right) + \left( {0.{445} \times {\ln}\left( {\text{wood density}} \right)} \right)} \right) \\ \end{aligned}$$

$$\begin{aligned} {\text{Evergreen forest }}\left( {{\text{TEFs I--III}}} \right){:}\;{\text{AGB}} & = {\exp}\left( {{1}.{662} - \left( {{1}.{114} \times {\ln}\left( {{\text{DBH}}} \right)} \right) + \left( {{1}.{169} \times \left( {{\ln}\left( {{\text{DBH}}} \right)} \right)^{{2}} \, } \right)} \right. \\ & \quad \left. { - \left( {0.{122} \times \left( {{\ln}\left( {{\text{DBH}}} \right)} \right)^{{3}} } \right) + \left( {0.{331} \times {\ln}\left( {\text{wood density}} \right)} \right)} \right) \\ \end{aligned}$$

AGB of bamboos was estimated following (Kaushal et al*.*^95^)

$${\text{AGB}} = 0.{1}00{2} \times \left( {\left( {{\text{DBH}}} \right)^{{{2}.{266}}} } \right)$$

AGB of palms was estimated following (Goodman et al*.*^60^)

$${\ln}\left( {{\text{AGB}}} \right) = - {3}.{3488} + \left( {{2}.{7483} \times {\ln}\left( {{\text{DBH}}} \right)} \right)$$

AGB of juvenile trees was estimated following (Chaturvedi et al*.*^96^)

$${\text{AGB}} = {3}.{344} + \left( {0.{443} \times \left( {{\ln}\left( {\left( {{\text{DBH}}} \right)^{{2}} } \right)} \right)} \right)$$

AGB of woody climbers was estimated following (Schnitzer et al*.*^97^)

$${\text{AGB}} = {\exp}\left( { - {1}.{484} + {2}.{657} \times \left( {{\ln}\left( {{\text{DBH}}} \right)} \right)} \right)$$

Belowground biomass (BGB) of adult trees was estimated following (Cairns et al*.*^98^)

$${\text{BGB}} = {\exp}\left( { - {1}.0{587} + 0.{8836} \times \left( {{\ln}\left( {{\text{AGB}}} \right)} \right)} \right)$$

BGB of juveniles, woody climbers, bamboos and palms was estimated following (Cairns et al*.*^98^ and Ravindranath and Ostwald^99^)

$${\text{BGB}} = {\text{AGB}} \times 0.{26}$$

Carbon content was estimated following (Junior et al*.*^100^)

$${\text{C}} = \left( {{\text{AGB}} + {\text{BGB}}} \right) \times 0.{4453}$$

### Understorey biomass C

The understorey (herbs and shrubs) biomass was determined using harvest method. Four 5 m × 5 m quadrats for shrubs and an equal number of 1 m × 1 m quadrats for herbs were laid randomly for biomass estimation. All understorey biomass falling within these quadrats was harvested during the peak growing season. The fresh weight of the harvested biomass was immediately measured with an electronic balance in situ and the collected representative samples were taken to the laboratory, oven-dried at 65 ± 5 °C for 72 h and weighed again for dry weight. Understorey C stock was then estimated by multiplying the dry weight with the corresponding C concentration.

### Litter and deadwood biomass C

The forest floor litter was collected from four replicates of 1 m × 1 m in each plot and segregated into leaves, fruits, flowers, twigs, bark and amorphous. All the fresh litter were weighed in situ and the collected representative samples were taken to the laboratory, oven-dried at 65 ± 5 °C for 48 h and weighed again for dry weight. Litter C was obtained by the multiplication of dry weight with its corresponding C concentration^101^

$${\text{Forest floor litter C}} = \left( {\text{Dry weight of litter}} \right) \times 0.{45}$$

In each plot, all the standing dead trees, fallen trees and cut stems were recorded for deadwood biomass C estimation^99^.

### Soil organic carbon

Soil samples were taken with a soil core sampler (internal diameter 5 cm) at six random points within each plot. The litter layer of the top soil was removed at the time of soil sampling. Soil samples were taken from each of the three depths separately (0–10, 10.1–20 and 20.1–30 cm) from each plot and three sets of composite soil samples were prepared for each plot. The composite soil samples were air-dried, sieved using a 2 mm mesh sieve and ground using a mortar and pestle to get fine particles. The SOC percent was estimated by Walkley and Black’s method^102^. The total C content of 0–30 cm depth was finally estimated by summing up the C content of all the three layers. The total SOC was calculated as (Pearson et al.^103^):

$${\text{SOC}}\left( {\text{Mg C/ha}} \right) = \left[ {\left( {{\text{Soil bulk density }}\left( {{\text{g/m}}^{{3}} } \right) \times {\text{Soil depth }}\left( {{\text{cm}}} \right) \times {\text{C}}} \right)} \right] \times {1}00$$

The total ecosystem-level C storage of each study site was calculated by summing up the C stocks of all the pools: total woody vegetation, understorey, litter, deadwood and soil.

### Environmental factors

The climatic variables such as minimum and maximum temperatures, mean annual temperature (MAT) and mean annual precipitation (MAP) were derived for all the plots from the WorldClim dataset^47^ using *sp* and *raster* packages in R 3.6.2^104–106^. The topographic variables such as slope and aspect were also derived using R 3.6.2^106^.

The disturbance factors were given a score of 0–6 calculated as the sum of values representing area impacted (0–3) and intensity (0–3). The factors were ranked based on the area impacted and intensity of influence as follows: 0 for none, 1 for low, 2 for medium and 3 for high. Scores of all the factors were summed to obtain a total disturbance score for each site (Supplementary Table S1)^107^.

### Data analyses

For each parameter, all values are presented as mean ± standard error. All statistical analyses were performed using SPSS 20.0^108^. Analysis of variance (ANOVA) with repeated measures (ANOVAR) was computed to compare the biomass and C stock values of different pools among and within the forest sites. Linear regression analyses were used to understand the relationships between C stocks of different components and predictor variables. Pearson correlation and Principal Components Analysis (PCA) were computed using PAST 3.0^109^ to understand the relationship of AGBC with diversity, structural attributes and environmental factors.

## Supplementary information

Supplementary Information.

## Data Availability

Data sets are given in the Supplementary information. Additional data used in this study are available from corresponding authors on request.
