# Supplementary material for: Ecosystem-level carbon storage and its links to diversity, structural and environmental drivers in tropical forests of Western Ghats, India
Source: Sci Rep. 2020 Aug 10;10:13444. doi: 10.1038/s41598-020-70313-6 (PMC7417561; doi:10.1038/s41598-020-70313-6)
Supplement: Supplementary file 1 — Supplementary Information. [file 41598_2020_70313_MOESM1_ESM.docx]

**Ecosystem-level carbon storage and its links to diversity, structural and environmental drivers in tropical forests of Western Ghats, India**

Subashree Kothandaraman^1+^, Javid Ahmad Dar^1+^, Somaiah Sundarapandian^1*^, Selvadurai Dayanandan^2*^, Mohammed Latif Khan^3*^

^1^Department of Ecology and Environmental Sciences, Pondicherry University, Puducherry-605014, India

^2^Centre for Structural and Functional Genomics, Biology Department, Concordia University, 7141 Sherbrooke St. W., Montreal H4B 1R6, Quebec, Canada

^3^Forest Ecology and Eco-genomics Laboratory, Department of Botany, Dr. Harisingh Gour Vishwavidyalaya (A Central University), Sagar, M.P., 470003, India

^*^email: smspandian65@gmail.com; khanml61@gmail.com; daya.dayanandan@concordia.ca

^+^K.S. and J.A.D. contributed equally to this study.


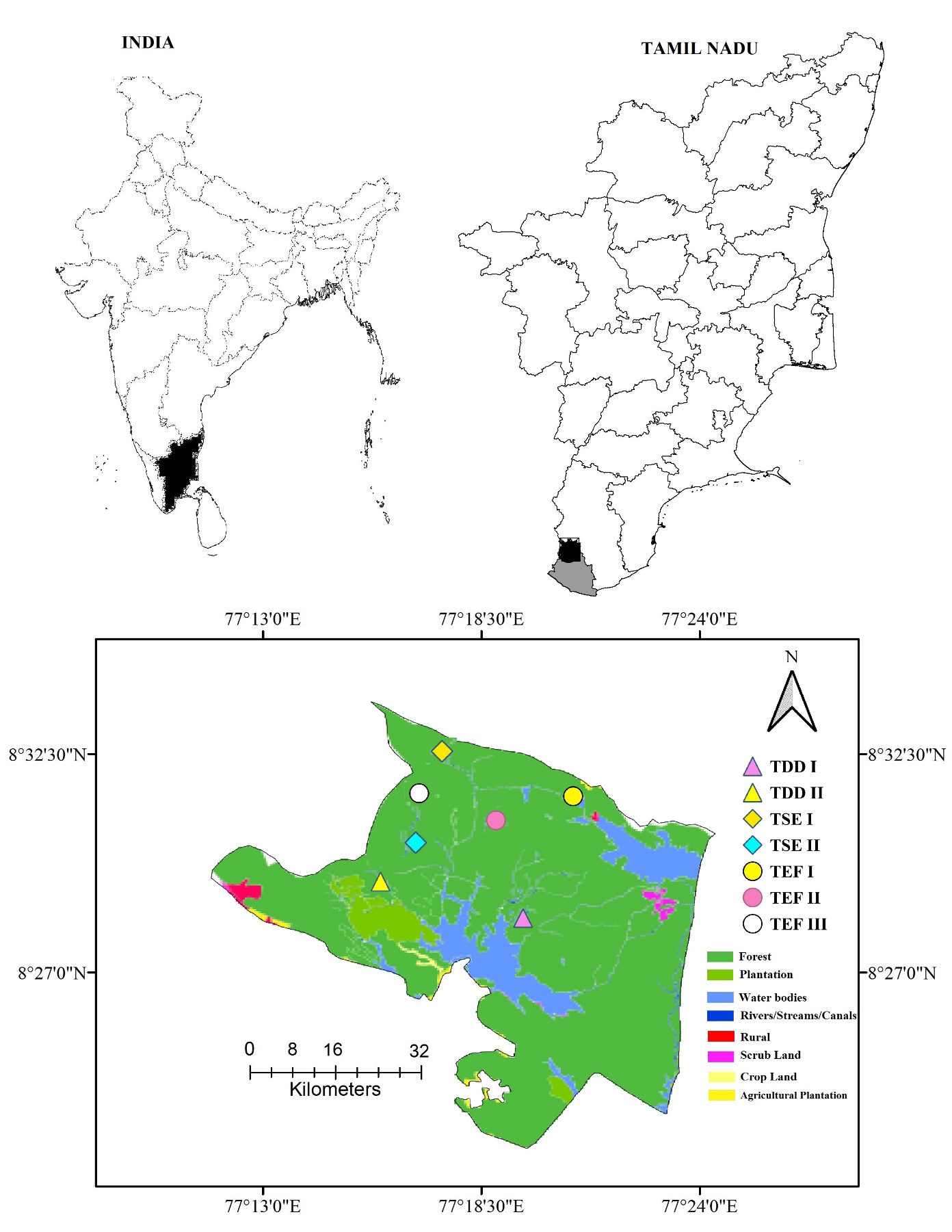


**Supplementary Figure S1.** Location of the seven study sites in Kanyakumari Wildlife Sanctuary, Western Ghats, India. Map was created using ArcMAP 10.2 https://desktop.arcgis.com/en/arcmap/

**Supplementary Figure S2a.** Relationships of juvenile and adult tree C stocks with (a,b) basal area (cm^2^/400 m^2^) (c,d) density (No./400 m^2^) and (e,f) species richness (No./plot)

**Species richness (No./plot)**

**Carbon stock (Mg C/ha)**

**Supplementary Figure S2b.** Relationships between tree species richness (No./plot) and C stock (Mg C/ha) (a) tree aboveground C (b) litter C (c) tree belowground C and (d) total ecosystem C

**Supplementary Table S1.** Site characteristics of seven forest sites in KWLS, Western Ghats, India

| **Variable** | **TDD I** | **TDD II** | **TSE I** | **TSE II** | **TEF I** | **TEF II** | **TEF III** |
| --- | --- | --- | --- | --- | --- | --- | --- |
| **Environmental factors** | | | | | | | |
| Mean annual precipitation (mm)^#^ | 1183 | 1310 | 1347 | 1310 | 1379 | 1379 | 1347 |
| Mean annual temperature (°C)^#^ | 26.8 | 25.7 | 24.6 | 25.3 | 24.3 | 24.6 | 24.6 |
| Minimum temperature (°C)^#^ | 21.6 | 20.5 | 19.3 | 20.1 | 19.0 | 19.3 | 19.3 |
| Maximum temperature (°C)^#^ | 32.3 | 31.1 | 30 | 30.8 | 29.8 | 30 | 30 |
| Mean elevation (m MSL) | 177.6 | 356.3 | 561.7 | 373.1 | 599.3 | 577.1 | 513.9 |
| Mean slope (°) | 5.0 | 9.1 | 7.6 | 11.3 | 13.1 | 9.6 | 7.6 |
| Mean aspect (°) | 202.9 | 272.0 | 203.6 | 181.3 | 249.4 | 226.0 | 203.6 |
| **Disturbance scores** | | | | | | | |
| Surrounding human settlements* | 6 | 0 | 0 | 0 | 0 | 0 | 0 |
| Grazing* | 4 | 0 | 0 | 0 | 0 | 0 | 0 |
| Ground fires* | 0 | 4 | 0 | 2 | 0 | 0 | 0 |
| Cut stems** | 4 | 0 | 0 | 0 | 2 | 0 | 0 |
| Invasion** | 4 | 0 | 0 | 0 | 0 | 0 | 0 |
| Total disturbance score | 18 | 4 | 0 | 2 | 2 | 0 | 0 |

^#^ - Data extracted from WorldClim (https://www.worldclim.org/)

* - Data based on enquiry with local people and personal observations of authors

** - Data based on field record

**Supplementary Table S2.** Comparison of biomass (Mg/ha) and carbon stocks (Mg C/ha) of different vegetation types recorded from the present and previous studies (AGB – aboveground biomass, AGC – aboveground carbon, TWC – total woody carbon, TVC – total vegetation carbon)

| **Vegetation type** | **Location** | **Area sampled (ha)** | **Size class** | **AGB (Mg/ha)** | **TWC**  **(Mg C/ha)** | **Ecosystem/**  **cumulative C**  **(Mg C/ha)** | **Source** |
| --- | --- | --- | --- | --- | --- | --- | --- |
| **TEMPERATE FOREST** | **Australia** |  |  |  |  |  |  |
| Managed forests | Kioloa, New South Wales | 6.12 | DBH ≥ 2 cm |  | 26.6-337.7  (mean 210.6, AGC) |  | Roxburgh *et al.*^1^ |
| Moist *Eucalyptus regnans* forests | O’Shannassy Catchment, Central Highlands of Victoria | 3.18 | DBH ≥ 5 cm |  | 104-1819  (mean 501, AGC) |  | Keith *et al.*^2^ |
|  | **North America** |  |  |  |  |  |  |
| Different vegetation types | Cofre de Perote volcano, Veracruz, Mexico | 1.75 | DBH ≥ 5 cm | 4.66-372 | 2.99-179.83 |  | Mendoza-Ponce and Galicia^3^ |
| Hard- and softwood forests | New England, USA | 4.8 | DBH ≥ 2.5 cm |  | 116, 125  (AGC) | 216, 267 (mean) | Hoover *et al.*^4^ |
|  | **Asia** |  |  |  |  |  |  |
| Different forest types | Mt. Changbai, China | 2.13 | DBH ≥ 5 cm |  | 52-245  (mean 153) | 112-338 (mean 237) | Zhu *et al.*^5^ |
| *Pinus tabulaeformis* forest | Liaoheyuan, Hebei Province, China | 2.16 |  | 101.3-314.4 | 62.4-177 | 178.1-359.8 | Zhao *et al.*^6^ |
| *Cedrus deodara* forest | Kumrat valley, Pakistan | 3.2 |  |  | 640.63 |  | Amir *et al.*^7^ |
| Korean pine mixed forest | Changbai Mountain Nature Reserve, China | 25 | DBH ≥ 1 cm | 269.3 |  |  | Yuan *et al.*^8^ |
| Community forest | Jamuna Danda Community  Forest, Kathmandu, Nepal | 0.75 | DBH ≥ 5 cm | 248.1 | 139.9 | 163 | Aryal *et al.*^9^ |
| Spruce forests | Gansu and Ningxia provinces, China | 3.9 | DBH ≥ 2 cm |  | 28.1-93.9  (mean 76.3) | 345.6-510.1 (mean 449.4) | Yue *et al.*^10^ |
|  | **India** |  |  |  |  |  |  |
| Different forest sites | Kedarnath Wildlife Sanctuary, Garhwal Himalaya, Uttarakhand | 1 | DBH ≥ 10 cm | 202.72-718.75 | 131.86-460.89 |  | Bhat *et al.*^11^ |
| Different forest types | Kashmir Himalaya, Jammu and Kashmir | 27.75 | GBH ≥ 10 cm | 79-237 (mean 187.6) | 45.4-135.6 | 112.5-205.7 | Dar and Sundarapandian^12^ |
| Different forest types | Sarwari Khad watershed, Western Himalaya, Himachal Pradesh |  | DBH ≥ 10 cm | 227.5-652.07 (mean 349.21) | 129.29-356.74  (mean 196.14) |  | Ghoshal and Samant^13^ |
| Coniferous and broad-leaved forests | Kashmir Himalaya, Jammu and Kashmir | 17.5 | DBH ≥ 3 cm | 71.8-227.6 |  |  | Dar *et al.*^14^ |
| **SUBTROPICAL/TROPICAL FOREST** | **Australia** |  |  |  |  |  |  |
| *Melaleuca* forest | Buckley’s Hole Conservation Park and Hays Inlet Conservation Park, Queensland | 0.9 | DBH ≥ 10 cm |  | 58.52-133.96 | 210.36-381.59 | Tran and Dargusch^15^ |
|  | **North America** |  |  |  |  |  |  |
| Semi-evergreen forest | Yucatan Peninsula, Mexico | 0.5 | DBH >1 and <10 cm | 33.5 |  |  | Cairns *et al.*^16^ |
| Semi-evergreen forest | Yucatan Peninsula, Mexico | 0.5 | DBH ≥ 10 cm | 191.5 |  |  | Cairns *et al.*^16^ |
| Montane forest | Cordillera Central, Dominican Republic | 7.5 | DBH ≥ 10 cm | 193-475 (mean 306) |  |  | Sherman *et al.*^17^ |
| Secondary semi-evergreen rainforest | “El Ocote” Biosphere Reserve, Mexico | 8.7 | DBH ≥ 10 cm |  | 24-104.9 | 115.4-211.5 | Orihuela-Belmonte *et al.*^18^ |
| Secondary forest | Calakmul Biosphere Reserve, Yucatan Peninsula, Mexico | 2.8 | DBH ≥ 1 cm |  | 15-123.39 | 91.77-231.67 | Aryal *et al.*^19^ |
| Mangrove forest | Atasta Peninsula, Campeche, Mexico | 0.24 | DBH ≥ 1 cm | 113.8-279.7 (mean 184.8) | 56.9-139.9  (mean 92.38, AGC) |  | Guerra-Santos *et al.*^20^ |
| Rainforest | Montes Azules Biosphere Reserve, Lacandon region, Mexico | 10.5 | DBH ≥ 10 cm |  | 155-374 | 287-478.1 | Navarrete-Segueda *et al.*^21^ |
|  | **Central America** |  |  |  |  |  |  |
| Moist forest | Barro Colorado Island, Panama | 50 | DBH ≥ 1 cm | 281 |  |  | Chave *et al.*^22^ |
| Montane forest | Central and South America | From several studies | DBH ≥ 10 cm | 78-408 (mean 224) |  |  | Lieberman *et al.*^23^, Delaney *et al.*^24^, Delaney *et al.*^25^, Spracklen and Righelato^26^, Leuschner *et al.*^27^, Girardin *et al.*^28^, Girardin *et al.*^29^, Moser *et al.*^30^, Unger *et al.*^31^, Álvarez-Arteaga *et al.*^32^, Nyirambangutse *et al.*^33^ |
|  | **South America** |  |  |  |  |  |  |
| *Terra-firme* forest | Central Amazonia, Brazil | 72 | DBH 1 - < 10 cm | 19.4 |  |  | de Castilho *et al.*^34^ |
| *Terra-firme* forest | Central Amazonia, Brazil | 72 | DBH 10 - < 30 cm | 109.3 |  |  | de Castilho *et al.*^34^ |
| *Terra-firme* forest | Central Amazonia, Brazil | 72 | DBH ≥ 30 cm | 198.3 |  |  | de Castilho *et al.*^34^ |
| Lowland forest | Amazonia | 227 plots | DBH ≥ 10 cm | 288.6 |  |  | Malhi *et al.*^35^ |
| Primary forest | Porce Region, Colombia | 3.3 | DBH ≥ 1 cm | 247.2 | 149.2 (TVC) | 252.4 | Sierra *et al.*^36^ |
| Secondary forest | Porce Region, Colombia | 3.85 | DBH ≥ 1 cm | 45.5 | 32.4 (TVC) | 107.9 | Sierra *et al.*^36^ |
| Moist forest | Atlantic forest, Brazil | 13 | DBH ≥ 4.8 cm | 166.3-283.2 |  |  | Alves *et al.*^37^ |
| Moist forest | Atlantic forest, Brazil | 13 | DBH > 10 cm | 154.8-271.7 |  |  | Alves *et al.*^37^ |
| Secondary forest | Andes, Colombia | 10 | DBH ≥ 10 cm | 104.85 |  |  | Pena and Duque^38^ |
| Lowland moist forest | South America | 33 locations | DBH ≥ 10 cm | 287.8 |  |  | Slik *et al.*^39^ |
| Dry forest | Gran Chaco, Argentina | 5 | DBH ≥ 5 cm |  | 0-22.13 | 32.99-85.27 | Conti *et al.*^40^ |
| Dry forest | Ceará, Brazil | 1 | Girth at base > 9 cm | 43.28 | 19.27 |  | Júnior *et al.*^41^ |
| Lowland forest types | Colombia | 4981 plots | DBH ≥ 10 cm | 226.9 |  |  | Phillips *et al.*^42^ |
| Lowland forest | Amazon, Magdalena and Orinoco River basins, Colombia | 32 | DBH > 10 cm | 145.66-405.84 |  |  | Aldana *et al.*^43^ |
| Different forest types | Northwest South America | 200 plots | ­ | 7.7-386.9 (mean 194.4) |  |  | Alvarez-Davila *et al.*^44^ |
| Lowland forest | Amazonia | From several studies | DBH ≥ 10 cm | 251-387 (mean 341) |  |  | Baker *et al.*^45^, Quesada *et al.*^46^, Nyirambangutse *et al.*^33^ |
| Humid forest | South America | 158 plots | DBH ≥ 10 cm |  | 133-148  (mean 140, AGC) |  | Sullivan *et al.*^47^ |
| Caatinga seasonally dry tropical  forest | Pernambuco state, Brazil | 1.8 | Diameter at ground level DGL > 3 cm |  | 15.74 (AGC, TVC) |  | Schulz *et al.*^48^ |
| Seasonal semi-deciduous forest | Minas Gerais, Brazil | 0.2 | GBH ≥ 15 cm | 126.9 | 55.91 (AGC) |  | Silva *et al.*^49^ |
| Evergreen slow-growth forest | Napo, Ecuador | 2 | DBH ≥ 10 cm | 246.8-320.9 |  |  | Torres *et al.*^50^ |
|  | **Africa** |  |  |  |  |  |  |
| Miombo woodland | Nhambita, Sofala Province, Mozambique | 27.2 | DBH ≥ 5 cm |  | 3.1-86.5  (mean 32.1) | 110 | Ryan *et al.*^51^ |
| Miombo woodland | Udzungwa mountain block, Eastern Arc Mountains, Tanzania | 4 | DBH ≥ 10 cm |  | 14.2-30.7  (mean 24.2, AGC) |  | Shirima *et al.*^52^ |
| Closed-canopy forest | Eastern Arc Mountains, Tanzania | 18 | DBH ≥ 10 cm |  | 144.8-205.5  (mean 174.6), 196.1-268.7 (mean 229.6, AGC, based on the equation used) |  | Marshall *et al.*^53^ |
| Miombo woodland | Zambia | 7.16 | DBH ≥ 5 cm |  | 28.7-52.8  (mean 39.6, AGC) |  | Kalaba *et al.*^54^ |
| Lowland forest | Gola, Upper Guinea, Sierra Leone | 76.13 | DBH ≥ 10 cm |  | 111.4-186.2  (mean 161.4, AGC) |  | Lindsell and Klop^55^ |
| Moist forest | Africa | 45 locations | DBH ≥ 10 cm | 418.3 |  |  | Slik *et al.*^39^ |
| Dry evergreen montane forest | Danaba community forest, Ethiopia | 3.32 | DBH ≥ 2.5 and < 5cm |  | 3.65 |  | Bazezew *et al.*^56^ |
| Dry evergreen montane forest | Danaba community forest, Ethiopia | 3.32 | DBH ≥ 5 cm |  | 315.78 |  | Bazezew *et al.*^56^ |
| Dry evergreen montane forest | Danaba community forest, Ethiopia | 3.32 | DBH ≥ 2.5 cm |  | 319.43 | 507.29 | Bazezew *et al.*^56^ |
| Moist forest | Lesio-louna forest, Republic of Congo | 2.76 | DBH ≥ 10 cm |  | 208.15 |  | Ekoungoulou *et al.*^57^ |
| Transitional rainforest | Kakamega forest, Kenya | 11.87 | DBH ≥ 5 cm | 77.7-1080.7 (mean 279.49) | 48.1-670  (mean 173.28) |  | Lung and Espira^58^ |
| Evergreen forests | Cameroon | 1050.5 | DBH ≥ 10 cm | 259.8 |  |  | Fayolle *et al.*^59^ |
| Evergreen forests | Cameroon | 1050.5 | DBH ≥ 20 cm | 213.1 |  |  | Fayolle *et al.*^59^ |
| Semi-deciduous forests | Cameroon | 2576 | DBH ≥ 10 cm | 347.6 |  |  | Fayolle *et al.*^59^ |
| Semi-deciduous forests | Cameroon | 2576 | DBH ≥ 20 cm | 281.8 |  |  | Fayolle *et al.*^59^ |
| Mistbelt forest | Limpopo Province, South Africa | 1.5 | DBH ≥ 5 cm | 358.1 | 179 (AGC) |  | Mensah *et al.*^60^ |
| Moist forest | Kakamega reserve forest, Kenya | 38.17 | DBH ≥ 5.1 cm |  | 59.3-345 (AGC) |  | Otuoma *et al.*^61^ |
| Deciduous miombo savanna woodland | Ngomakurira Mountain, Domboshawa, Zimbabwe | 0.52 | DBH ≥ 8 cm | 34.5-65.1 |  |  | Zimudzi and Chapano^62^ |
| Dry forest | Banja forest, Ethiopia | 2.52 | DBH ≥ 5 cm | 720.69 | 406.46  (mean 338.72) | 639.87 | Abere *et al.*^63^ |
| Different forest types | Mt. Nyiro, Mt. Kulal and Mt. Marsabit, Kenya | 4.8 | DBH ≥ 10 cm | 117.5-611.8 |  |  | Cuni-Sanchez *et al.*^64^ |
| Montane forest | Central and East Africa | From several studies | DBH ≥ 10 cm | 275-380 (mean 327) |  |  | Hemp^65^, Ensslin *et al.*^66^, Rutten *et al.*^67^, Nyirambangutse *et al.*^33^ |
| Lowland forest | Central Africa | 312.5 | DBH ≥ 10 cm | 147-749 (mean 429) |  |  | Lewis *et al.*^68^, Nyirambangutse *et al.*^33^ |
| Miombo woodland | Kilwa, Lindi, Tanzania | 25 | DBH ≥ 5 cm |  | 2-54  (mean 24, AGC) |  | McNicol *et al.*^69^ |
| Montane forest | Nyungwe National Park, Rwanda | 7.5 | DBH ≥ 5 cm | 274-279 | 86.9-460  (mean 163) | 232-662 (353) | Nyirambangutse *et al.*^33^ |
| Montane forest | Nyungwe National Park, Rwanda | 2.5 | DBH ≥ 10 cm | 380 |  |  | Nyirambangutse *et al.*^33^ |
| Dry forest | Gergera watershed, Eastern Tigray, Ethiopia | 0.54 | DBH ≥ 2 cm |  | 5.75-44.71 |  | Solomon *et al.*^70^ |
| Humid forest | Africa | 162 plots | DBH ≥ 10 cm |  | 176-190  (mean 183, AGC) |  | Sullivan *et al.*^47^ |
| Savannah | Plateau Batéké, Congo | 4 | DBH ≥ 5 cm | 9.17 |  |  | Ifo *et al.*^71^ |
| Different vegetation types | W National Park, Tapoa, Burkina Faso | 8.8 | DBH ≥ 5 cm | 6.21-122.79 | 2.92-57.71 (AGC) |  | Dimobe *et al.*^72^ |
| Guinea natural forest | Ekiti, Nigeria | 0.3 | DBH ≥ 5 cm | 251.2 | 145.83 | 155.61 | Olorunfemi *et al.*^73^ |
|  |  |  |  |  |  |  |  |
|  | **Asia (except India)** |  |  |  |  |  |  |
| Different forest types | Thong Pha Phum National Forest, Kanchanaburi Province, Thailand | 5.73 | DBH ≥ 4.5 cm | 96.28-275.46 | 48.14-137.73 (AGC) |  | Terakunpisut *et al.*^74^ |
| Seasonal rainforest | Xishuangbanna, China | 3 | DBH ≥ 2 cm |  | 163.8-259.8  (mean 199.9, live only) | 260.5-377.4 (mean 303.3) | Lü *et al.*^75^ |
| Lowland dipterocarp rainforest | Sabah, Malaysian Borneo | 2 | DBH ≥ 10 cm |  | 108.4 | 150.3 | Saner *et al.*^76^ |
| Lowland dipterocarp rainforest | Sabah, Malaysian Borneo | 2 | DBH ≥ 10 cm |  | 108.4 | 167.9 | Saner *et al.*^76^ |
| Forest over limestone | Xishuangbanna, Yunnan Province, China | 1 | DBH ≥ 2 cm |  | 116.35-220.73  (mean 158.52) | 173.42-294.32 (mean 213.76) | Tang *et al.*^77^ |
| Natural forest | Tankawati natural hill forest, Bangladesh | 3.2 |  | 182.48 | 110.94 | 283.8 | Ullah and Al-Amin^78^ |
| Primary forest | Bukit Timah Nature Reserve, Singapore |  |  |  | 167.5 (AGC) | 336.7 | Ngo *et al.*^79^ |
| Secondary forest | Bukit Timah Nature Reserve, Singapore |  |  |  | 104.5 | 274.2 | Ngo *et al.*^79^ |
| Moist forest | Southeast Asia | 42 locations | DBH ≥ 10 cm | 393.3 |  |  | Slik *et al.*^39^ |
| Montane and lowland forests | Pahang, Malaysia | 1.8 | DBH ≥ 5 cm |  | 92.5-147.7 | 113.8-255.03 | Jeyanny *et al.*^80^ |
| Broadleaved evergreen forest | Pearl River Delta, Guangdong Province, China | 1.92 | Height > 3 m |  | 40.17-154.05 | 125.23-288.88 | Sun and Guan^81^ |
| Mangrove forest | Yingluo Bay, China | 0.92 |  | 65.21-114.5 | 42.07-67.01 |  | Wang *et al.*^82^ |
| Sal forest | Terai Arc Landscape, Nepal | 5.65 | DBH ≥ 5 cm |  | 0.29-439.83  (mean 105.6, AGC) | 228.76 (mean) | Gurung *et al.*^83^ |
| Evergreen broadleaf forest | Central Highland, Vietnam | 37.5 | DBH ≥ 5 cm | 18.1-359.7 |  | 75.2-254.8 | Hai *et al.*^84^ |
| Semi-evergreen forest | Cambodia | 17.9 | DBH ≥ 10 cm |  | 95.8-102.2  (mean 99.8, AGC) |  | Chheng *et al.*^85^ |
| Different forest types and plantation | Misamis Occidental, Philippines | 1.2 | DBH ≥ 5 cm | 192.56-641.04 (mean 371.45) | 86.65-288.47  (mean 167.15) | 159.7-400.6 (mean 242.28) | Ebasan *et al.*^86^ |
| Secondary forest | Southern Anhui Province, China |  | DBH ≥ 7 cm |  | 114 (TVC) | 318 | Fan *et al.*^87^ |
| Different forest types | Central and southern Sri Lanka | 17.28 | DBH ≥ 3 cm |  | 22-181 /19-146 (AGC, based on the equation) |  | Mattsson *et al.*^88^ |
| Sal forest | Bishnunagar community forest, Nawalparasi, Nepal | 1.2 | DBH ≥ 5 cm |  | 115.7 |  | Pathak and Baniya^89^ |
| Montane forest | Southeast Asia | From several studies | DBH ≥ 10 cm | 119-307 (mean 248) |  |  | Edwards and Grubb^90^, Kitayama and Aiba^91^, Aiba *et al.*^92^, Culmsee *et al.*^93^, Dossa *et al.*^94^, Sawada *et al.*^95^, Nyirambangutse *et al.*^33^ |
| Fengshui forests | Fengshui forests, Guangzhou, China | 7.2 |  |  | 85.4-153 (AGC) | 242.1-399 | Ma *et al.*^96^ |
| Lowland forest | Borneo | 83 tree inventories | DBH ≥ 10 cm | 196-779 (mean 457.1) |  |  | Slik *et al.*^97^, Nyirambangutse *et al.*^33^ |
| Humid forest | Southeast Asia | 40 plots | DBH ≥ 10 cm |  | 180-215  (mean 197, AGC) |  | Sullivan *et al.*^47^ |
| Different forest types | Zhejiang Province, China | 50.28 | DBH ≥ 5 cm |  | 27.34 | 145.22 | Dai *et al.*^98^ |
| Mixed evergreen broad-leaved forest | Gutianshan National  Nature Reserve, Zhejiang Province, China | 22.5 | DBH ≥ 10 cm |  | 57.4 (AGC) | 149.2 | Liu *et al.*^99^ |
| Evergreen forest | K’Bang district, Gia Lai  province, Vietnam | 6 | DBH ≥ 10 cm | 327.2 | 177.4 | 324-393 (mean 355.4) | Nam *et al.*^100^ |
| Different forest types | Liaoning Province, China | 6.52 | DBH ≥ 2 cm |  | 28.99 (AGC) | 183.57 | Wang *et al.*^101^ |
|  | **India (except Eastern and Western Ghats)** |  |  |  |  |  |  |
| Dry deciduous forest | Tamil Nadu | 10 | GBH ≥ 10 cm | 39.69-170.02/73.06-173.10 (based on the equation) |  |  | Mani and Parthasarathy^102^ |
| Dry deciduous mixed and dry sal forests | Chhindwara, Satpura plateau, Madhya Pradesh | 0.8 |  | 154.9-345.6 |  |  | Pande and Patra^103^ |
| Dry deciduous forest | Uttar Pradesh | 4.5 | DBH ≥ 9.5 cm |  | 15.6-151  (mean 87, AGC) |  | Chaturvedi *et al.*^104^ |
| Dry forest | Sacred groves, Tamil Nadu | 4 | GBH ≥ 30 cm | 58.43-102.76 | 33.93-58.99 |  | Sundarapandian *et al.*^105^ |
| Moist deciduous forest | Sal forest, Goalpara, Assam | 0.5 | DBH ≥ 10 cm | 239.45 | 119.73 (AGC) |  | Rabha^106^ |
| Evergreen and deciduous forests | Gibbon Wildlife Sanctuary and Kholahat reserve forest, Assam |  | DBH ≥ 10 cm | 135.29-146.42 | 67.65-73.21 (AGC) |  | Borah *et al.*^107^ |
| Natural forest types and teak plantation | Barnawapara Wildlife Sanctuary, Chhatisgarh |  |  | 112.73-414.26 | 58.06-208.22 |  | Lal *et al.*^108^ |
| Different forest types | Trishna Wildlife Sanctuary, Tripura | 10 | GBH ≥ 10 cm | 37.85-85.59 | 18.93-42.8 (AGC) |  | Majumdar *et al.*^109^ |
| Dry deciduous forest | Madhya Pradesh | 4.8 | DBH ≥ 10 cm | 13-54 | 6-26 (AGC) |  | Salunkhe and Khare^110^ |
| Dry deciduous forest | Gurgaon district, Haryana | 0.48 | GBH > 31.5 cm | 37.93-63.73 | 25.33-42.48 |  | Singh *et al.*^111^ |
| Dry forest | Jharkhand | 0.2 |  |  | 1.84-231.18 | 8-273 | Ahirwal and Maiti^112^ |
| Moist deciduous forest | Katerniaghat Wildlife Sanctuary, Uttar Pradesh | 9 | DBH ≥ 5 cm | 290.82-455.99 | 141.18-228.87 (AGC) |  | Behera *et al.*^113^ |
| Wet evergreen rainforest | Jeypore reserve forest, Assam | 0.6 | DBH ≥ 5 cm | 101.26-282.61 | 66.83-186.53 | 102.43-306.61 | Gogoi *et al.*^114^ |
| Floodplain forest | Chatla floodplain, Assam | 0.2 |  | 552.33 | 263.2 (AGC) |  | Nath *et al.*^115^ |
| Different forest types | Senapati, Manipur | 0.4 | DBH ≥ 10 cm |  | 25.59-164.81 (AGC) |  | Niirou and Gupta^116^ |
| Different forest sites | Manipur | 4 | DBH ≥ 10 cm | 121.5-253.9 | 60.09-121.43 (AGC) |  | Thokchom and Yadava^117^ |
|  | **Eastern Ghats** |  |  |  |  |  |  |
| Different forest types | Kolli hills, Tamil Nadu | 5 | GBH ≥ 30 cm | 57.5-307.3 |  |  | Ramachandran *et al.*^118^ |
| Different forest types | Kolli hills, Tamil Nadu | 1.63 | DBH ≥ 30 cm | 15.61-597.13 | 7.8-298.56  (mean 170.7, AGC) |  | Mohanraj *et al.*^119^ |
| Dry forest | Deogarh district, Odisha | 5.12 | DBH ≥ 15 cm | 13.96-514.5 (mean 98.87) |  |  | Sahu *et al.*^120^ |
| Dry deciduous forest | Sathanur reserve forest | 30 | GBH ≥10 to < 30 cm | 0.39-3.37 (mean 1.86) |  |  | Gandhi and Sundarapandian^121^ |
| Dry deciduous forest | Sathanur reserve forest | 30 | GBH ≥ 30 cm | 64.81-624.96 (mean 245.9) |  |  | Gandhi and Sundarapandian^121^ |
| Dry deciduous forest | Sathanur reserve forest | 30 |  |  | 37.86-322.16  (mean 131.38) |  | Gandhi and Sundarapandian^121^ |
| Dry forest | Javadi hills | 3.75 | GBH ≥10 to < 30 cm | 1.78-4.29 |  |  | Naveenkumar *et al.*^122^ |
| Dry forest | Javadi hills | 3.75 | GBH ≥ 30 cm | 94.85-214.44 |  |  | Naveenkumar *et al.*^122^ |
| Dry forest | Javadi hills | 3.75 |  |  | 53.07-116.06 |  | Naveenkumar *et al.*^122^ |
|  |  |  |  |  |  |  |  |
|  | **Western Ghats** |  |  |  |  |  |  |
| Different forest types | Kanyakumari Wildlife Sanctuary, Tamil Nadu | 2.8 | DBH 3-9.9 cm | 1.23-6.07 (mean 4.19) |  |  | Present study |
| Different forest types | Kanyakumari Wildlife Sanctuary, Tamil Nadu | 2.8 | DBH ≥ 10 cm | 160.8-868.2 (mean 443.2) |  |  | Present study |
| Different forest types | Kanyakumari Wildlife Sanctuary, Tamil Nadu | 2.8 |  |  | 89.6-445.7  (mean 231.3) | 226.8-513.7 (mean 336.8) | Present study |
| Wet evergreen forest | Kodagu and North Kanara, Karnataka | 1.28 | DBH ≥ 30 cm | 378.5-507.6 |  |  | Swamy *et al.*^123^ |
| Evergreen and deciduous forests | Uttara Kannada, Karnataka | 12 | GBH ≥ 10 cm | 344-417 | 165, 177 |  | Murthy *et al.*^124^ |
| Evergreen forest | Uppangala, Karnataka | 6 | GBH ≥ 30 cm | 268.05-649.82 (mean 478.74) |  |  | Jeyakumar *et al.*^125^ |

**References**

1. Roxburgh, S.H., Wood, S.W., Mackey, B.G., et al., 2006. Assessing the carbon sequestration potential of managed forests: a case study from temperate Australia. Journal of Applied Ecology, 43(6), pp.1149-1159.
2. Keith, H., Mackey, B.G. and Lindenmayer, D.B., 2009. Re-evaluation of forest biomass carbon stocks and lessons from the world's most carbon-dense forests. Proceedings of the National Academy of Sciences, 106(28), pp.11635-11640.
3. Mendoza-Ponce, A. and Galicia, L., 2010. Aboveground and belowground biomass and carbon pools in highland temperate forest landscape in Central Mexico. Forestry, 83(5), pp.497-506.
4. Hoover, C.M., Leak, W.B. and Keel, B.G., 2012. Benchmark carbon stocks from old-growth forests in northern New England, USA. Forest Ecology and Management, 266, pp.108-114.
5. Zhu, B., Wang, X., Fang, J., et al., 2010. Altitudinal changes in carbon storage of temperate forests on Mt Changbai, Northeast China. Journal of Plant Research, 123(4), pp.439-452.
6. Zhao, J., Kang, F., Wang, L., et al., 2014. Patterns of biomass and carbon distribution across a chronosequence of Chinese pine (*Pinus tabulaeformis*) forests. PLoS One, 9(4), p.e94966.
7. Amir, M., Khan, A., Ahmad, A., et al., 2015. Carbon Stocks of Pure *Cedrus Deodara* Forest in Kumrat Valley, Dir Upper, KPK, Pakistan. International Journal of Scientific & Engineering Research, 6(4), pp. 1146-1150.
8. Yuan, Z., Gazol, A., Wang, X., et al., 2016. Pattern and dynamics of biomass stock in old growth forests: The role of habitat and tree size. Acta Oecologica, 75, pp.15-23.
9. Aryal, S., Shrestha, S., Maraseni, T., et al., 2018. Carbon stock and its relationships with tree diversity and density in community forests in Nepal. International Forestry Review 20(3), pp. 263-273.
10. Yue, J.W., Guan, J.H., Deng, L., et al., 2018. Allocation pattern and accumulation potential of carbon stock in natural spruce forests in northwest China. PeerJ 6:e4859
11. Bhat, J.A., Iqbal, K., Kumar, M., et al., 2013. Carbon stock of trees along an elevational gradient in temperate forests of Kedarnath Wildlife Sanctuary. Forest Science and Practice, 15(2), pp.137-143.
12. Dar, J.A. and Sundarapandian, S., 2015. Variation of biomass and carbon pools with forest type in temperate forests of Kashmir Himalaya, India. Environmental Monitoring and Assessment, 187(2), p.55.
13. Ghoshal, S. and Samant, S.S., 2015. Assessment of Tree Carbon Stocks of Forests: A Case Study of the Sarwari Khad Watershed, Western Himalaya, India. Environment & We: An International Journal of Science & Technology 10, 51-61.
14. Dar, J.A., Rather, M.Y., Subashree, K., et al., 2017. Distribution patterns of tree, understorey, and detritus biomass in coniferous and broad-leaved forests of Western Himalaya, India. Journal of Sustainable Forestry, 36(8), pp.787-805.
15. Tran, D.B. and Dargusch, P., 2016. Melaleuca forests in Australia have globally significant carbon stocks. Forest Ecology and Management, 375, pp.230-237.
16. Cairns, M.A., Olmsted, I., Granados, J. et al., 2003. Composition and aboveground tree biomass of a dry semi-evergreen forest on Mexico’s Yucatan Peninsula. Forest Ecology and Management, 186(1-3), pp.125-132.
17. Sherman, R.E., Fahey, T.J., Martin, P.H. et al., 2012. Patterns of growth, recruitment, mortality and biomass across an altitudinal gradient in a neotropical montane forest, Dominican Republic. Journal of Tropical Ecology, 28(5), pp.483-495.
18. Orihuela-Belmonte, D.E., De Jong, B.H.J., Mendoza-Vega, J., et al., 2013. Carbon stocks and accumulation rates in tropical secondary forests at the scale of community, landscape and forest type. Agriculture, Ecosystems & Environment, 171, pp.72-84.
19. Aryal, D.R., de Jong, B.H., Ochoa-Gaona, S., et al., 2014. Carbon stocks and changes in tropical secondary forests of southern Mexico. Agriculture, Ecosystems & Environment, 195, pp.220-230.
20. Guerra-Santos, J.J., Cerón-Bretón, R.M., Cerón-Bretón, J.G., et al., 2014. Estimation of the carbon pool in soil and above-ground biomass within mangrove forests in Southeast Mexico using allometric equations. Journal of Forestry Research, 25(1), pp.129-134.
21. Navarrete-Segueda, A., Martínez-Ramos, M., Ibarra-Manríquez, G., et al., 2018. Variation of main terrestrial carbon stocks at the landscape-scale are shaped by soil in a tropical rainforest. Geoderma, 313, pp.57-68.
22. Chave, J., Condit, R., Lao, S., et al., 2003. Spatial and temporal variation of biomass in a tropical forest: results from a large census plot in Panama. Journal of Ecology, 91(2), pp.240-252.
23. Lieberman, D., Lieberman, M., Peralta, R. et al., 1996. Tropical forest structure and composition on a large-scale altitudinal gradient in Costa Rica. Journal of Ecology, 84(2),pp.137-152.
24. Delaney, M., Brown, S., Lugo, A.E., et al., 1997. The distribution of organic carbon in major components of forests located in five life zones of Venezuela. Journal of Tropical Ecology, 13(5), pp.697-708.
25. Delaney, M., Brown, S., Lugo, A.E., et al., 1998. The quantity and turnover of dead wood in permanent forest plots in six life zones of Venezuela. Biotropica, 30(1), pp.2-11.
26. Spracklen, D.V. and Righelato, R., 2005. Carbon storage and sequestration in tropical montane forests of southern Ecuador. World Land Trust, Halesworth, Suffolk, UK.
27. Leuschner, C., Moser, G., Bertsch, C., et al., 2007. Large altitudinal increase in tree root/shoot ratio in tropical mountain forests of Ecuador. Basic and Applied Ecology, 8(3), pp.219-230.
28. Girardin, C.A.J., Malhi, Y., Aragao, L.E.O.C., et al., 2010. Net primary productivity allocation and cycling of carbon along a tropical forest elevational transect in the Peruvian Andes. Global Change Biology, 16(12), pp.3176-3192.
29. Girardin, C.A., Farfan-Rios, W., Garcia, K., et al., 2014. Spatial patterns of above-ground structure, biomass and composition in a network of six Andean elevation transects. Plant Ecology & Diversity, 7(1-2), pp.161-171.
30. Moser, G., Leuschner, C., Hertel, D., et al., 2011. Elevation effects on the carbon budget of tropical mountain forests (S Ecuador): the role of the belowground compartment. Global Change Biology, 17(6), pp.2211-2226.
31. Unger, M., Homeier, J. and Leuschner, C., 2012. Effects of soil chemistry on tropical forest biomass and productivity at different elevations in the equatorial Andes. Oecologia, 170(1), pp.263-274.
32. Alvarez-Arteaga, G., García-Calderón, N.E., Krasilnikov, P. et al., 2013. Carbon storage in montane cloud forests in Sierra Norte of Oaxaca, México. Agrociencia (Montecillo), 47(2), pp.171-180.
33. Nyirambangutse, B., Zibera, E., Uwizeye, F.K., et al., 2017. Carbon stocks and dynamics at different successional stages in an Afromontane tropical forest. Biogeosciences, 14(5), pp.1285-1303.
34. de Castilho, C.V., Magnusson, W.E., de Araújo, R.N.O., et al., 2006. Variation in aboveground tree live biomass in a central Amazonian Forest: Effects of soil and topography. Forest Ecology and Management, 234(1-3), pp.85-96.
35. Malhi, Y., Wood, D., Baker, T.R., et al., 2006. The regional variation of aboveground live biomass in old‐growth Amazonian forests. Global Change Biology, 12(7), pp.1107-1138.
36. Sierra, C.A., del Valle, J.I., Orrego, S.A., et al., 2007. Total carbon stocks in a tropical forest landscape of the Porce region, Colombia. Forest Ecology and Management, 243(2-3), pp.299-309.
37. Alves, L.F., Vieira, S.A., Scaranello, M.A., et al., 2010. Forest structure and live aboveground biomass variation along an elevational gradient of tropical Atlantic moist forest (Brazil). Forest Ecology and Management, 260(5), pp.679-691.
38. Peña, M.A. and Duque, A., 2013. Patterns of stocks of aboveground tree biomass, dynamics, and their determinants in secondary Andean forests. Forest Ecology and Management, 302, pp.54-61.
39. Slik, J.W., Paoli, G., McGuire, K., et al., 2013. Large trees drive forest aboveground biomass variation in moist lowland forests across the tropics. Global Ecology and Biogeography, 22(12), pp.1261-1271.
40. Conti, G., Pérez-Harguindeguy, N., Quètier, F., et al., 2014. Large changes in carbon storage under different land-use regimes in subtropical seasonally dry forests of southern South America. Agriculture, Ecosystems & Environment, 197, pp.68-76.
41. Júnior, P., Resende, L., Andrade, E.M.D., et al., 2016. Carbon stocks in a tropical dry forest in Brazil. Revista Ciência Agronômica, 47(1), pp.32-40.
42. Phillips, J., Duque, Á., Scott, C., et al., 2016. Live aboveground carbon stocks in natural forests of Colombia. Forest Ecology and Management, 374, pp.119-128.
43. Aldana, A.M., Villanueva, B., Cano, Á., et al., 2017. Drivers of biomass stocks in Northwestern South American forests: Contributing new information on the Neotropics. Forest Ecology and Management, 389, pp.86-95.
44. Álvarez-Davila, E., Cayuela, L., González-Caro, S., et al., 2017. Forest biomass density across large climate gradients in northern South America is related to water availability but not with temperature. PLoS One, 12(3), p.e0171072.
45. Baker, T.R., Phillips, O.L., Malhi, Y., et al., 2004. Variation in wood density determines spatial patterns in Amazonian forest biomass. Global Change Biology, 10(5), pp.545-562.
46. Quesada, C.A., Lloyd, J., Schwarz, M., et al., 2010. Variations in chemical and physical properties of Amazon forest soils in relation to their genesis. Biogeosciences, 7(5), pp.1515-1541.
47. Sullivan, M.J., Talbot, J., Lewis, S.L., et al., 2017. Diversity and carbon storage across the tropical forest biome. Scientific Reports, 7, p.39102.
48. Schulz, K., Guschal, M., Kowarik, I., et al., 2018. Grazing, forest density, and carbon storage: towards a more sustainable land use in Caatinga dry forests of Brazil. Regional Environmental Change, <https://doi.org/10.1007/s10113-018-1303-0>
49. Silva, H.F., Ribeiro, S.C., Botelho, S.A., et al., 2018. Biomass and Carbon in a Seasonal Semideciduous Forest in Minas Gerais. Floresta e Ambiente, 25(1): e20160508.
50. Torres, B., Vasseur, L., Lopez, R., et al., 2019. Structure and above ground biomass along an elevation small-scale gradient: case study in an Evergreen Andean Amazon forest, Ecuador. Agroforestry systems, <https://doi.org/10.1007/s10457-018-00342-8>
51. Ryan, C.M., Williams, M. and Grace, J., 2011. Above- and Belowground Carbon Stocks in a Miombo Woodland Landscape of Mozambique. Biotropica, 43(4): 423–432.
52. Shirima, D.D., Munishi, P.K., Lewis, S.L., et al., 2011. Carbon storage, structure and composition of miombo woodlands in Tanzania’s Eastern Arc Mountains. African Journal of Ecology, 49(3), pp.332-342.
53. Marshall, A.R., Willcock, S., Platts, P.J., et al., 2012. Measuring and modelling above-ground carbon and tree allometry along a tropical elevation gradient. Biological Conservation, 154, pp.20-33.
54. Kalaba, F.K., Quinn, C.H., Dougill, A.J. et al., 2013. Floristic composition, species diversity and carbon storage in charcoal and agriculture fallows and management implications in Miombo woodlands of Zambia. Forest Ecology and Management, 304, pp.99-109.
55. Lindsell, J.A. and Klop, E., 2013. Spatial and temporal variation of carbon stocks in a lowland tropical forest in West Africa. Forest Ecology and Management, 289, pp.10-17.
56. Bazezew, M.N., Soromessa, T. and Bayable, E., 2015. Carbon stock in Adaba-Dodola community forest of Danaba District, West-Arsi zone of Oromia Region, Ethiopia: An implication for climate change mitigation. Journal of Ecology and The Natural Environment, 7(1), pp.14-22.
57. Ekoungoulou, R., Niu, S., Loumeto, J.J., et al., 2015. Evaluating the carbon stock in above-and below-ground biomass in a moist central African forest. Applied Ecology and Environmental Sciences, 3(2), pp.51-59.
58. Lung, M. and Espira, A., 2015. The influence of stand variables and human use on biomass and carbon stocks of a transitional African forest: Implications for forest carbon projects. Forest Ecology and Management, 351, pp.36-46.
59. Fayolle, A., Panzou, G.J.L., Drouet, T., et al., 2016. Taller trees, denser stands and greater biomass in semi-deciduous than in evergreen lowland central African forests. Forest Ecology and Management, 374, pp.42-50.
60. Mensah, S., Veldtman, R., Du Toit, B., et al., 2016. Aboveground biomass and carbon in a South African mistbelt forest and the relationships with tree species diversity and forest structures. Forests, 7(4), p.79.
61. Otuoma, J., Anyango, B., Ouma, G., et al., 2016. Determinants of aboveground carbon offset additionality in plantation forests in a moist tropical forest in western Kenya. Forest Ecology and Management, 365, pp.61-68.
62. Zimudzi, C. and Chapano, C., 2016. Diversity, Population Structure, and Above Ground Biomass in Woody Species on Ngomakurira Mountain, Domboshawa, Zimbabwe. International Journal of Biodiversity, 2016, p.4909158.
63. Abere, F., Belete, Y., Kefalew, A. et al., 2017. Carbon stock of Banja forest in Banja district, Amhara region, Ethiopia: An implication for climate change mitigation. Journal of Sustainable Forestry, 36(6), pp.604-622.
64. Cuni-Sanchez, A., Pfeifer, M., Marchant, R., et al., 2017. New insights on above ground biomass and forest attributes in tropical montane forests. Forest Ecology and Management, 399, pp.235-246.
65. Hemp, A., 2006. Continuum or zonation? Altitudinal gradients in the forest vegetation of Mt. Kilimanjaro. Plant Ecology, 184(1), pp.27-42.
66. Ensslin, A., Rutten, G., Pommer, U., et al., 2015. Effects of elevation and land use on the biomass of trees, shrubs and herbs at Mount Kilimanjaro. Ecosphere, 6(3), pp.1-15.
67. Rutten, G., Ensslin, A., Hemp, A. et al., 2015. Forest structure and composition of previously selectively logged and non-logged montane forests at Mt. Kilimanjaro. Forest Ecology and Management, 337, pp.61-66.
68. Lewis, S.L., Sonké, B., Sunderland, T., et al., 2013. Above-ground biomass and structure of 260 African tropical forests. Philosophical Transactions of the Royal Society B: Biological Sciences, 368(1625), p.20120295.
69. McNicol, I.M., Ryan, C.M., Dexter, K.G., et al., 2017. Aboveground Carbon Storage and Its Links to Stand Structure, Tree Diversity and Floristic Composition in South-Eastern Tanzania. Ecosystems, pp.1-15. DOI: 10.1007/s10021-017-0180-6
70. Solomon, N., Birhane, E., Tadesse, T., et al., 2017. Carbon stocks and sequestration potential of dry forests under community management in Tigray, Ethiopia. Ecological Processes, 6(1), p.20.
71. Ifo, A.S., Gomat, H.Y., Wenina, Y.E.M., et al., 2018. Carbon stocks and tree allometries in the savannahs of the Plateau Batéké, central Africa. Forest Ecology and Management 427, pp.86–95.
72. Dimobe, K., Kuyah, S., Dabré, Z., et al., 2019. Diversity-carbon stock relationship across vegetation types in W National park in Burkina Faso. Forest Ecology and Management 438, pp.243–254.
73. Olorunfemi, I.E., Komolafe, A.A., Fasinmirin, J.T., et al., 2019. Biomass carbon stocks of different land use management in the forest vegetative zone of Nigeria. Acta Oecologica 95, pp.45–56.
74. Terakunpisut, J., Gajaseni, N. and Ruankawe, N., 2007. Carbon sequestration potential in aboveground biomass of Thong Pha Phum national forest, Thailand. Applied Ecology and Environmental Research, 5(2), pp.93-102.
75. Lü, X.T., Yin, J.X., Jepsen, M.R. et al., 2010. Ecosystem carbon storage and partitioning in a tropical seasonal forest in Southwestern China. Forest Ecology and Management, 260(10), pp.1798-1803.
76. Saner, P., Loh, Y.Y., Ong, R.C. et al., 2012. Carbon stocks and fluxes in tropical lowland dipterocarp rainforests in Sabah, Malaysian Borneo. PLoS One, 7(1), p.e29642.
77. Tang, J.W., Yin, J.X., Qi, J.F., et al., 2012. Ecosystem carbon storage of tropical forests over limestone in Xishuangbanna, SW China. Journal of Tropical Forest Science, pp.399-407.
78. Ullah, M.R. and Al-Amin, M., 2012. Above-and below-ground carbon stock estimation in a natural forest of Bangladesh. Journal of Forest Science, 58(8), pp.372-379.
79. Ngo, K.M., Turner, B.L., Muller-Landau, H.C., et al., 2013. Carbon stocks in primary and secondary tropical forests in Singapore. Forest Ecology and Management, 296, pp.81-89.
80. Jeyanny, V., Husni, M.H.A., Rasidah, K.W., et al., 2014. Carbon stocks in different carbon pools of a tropical lowland forest and a montane forest with varying topography. Journal of Tropical Forest Science, pp.560-571.
81. Sun, L. and Guan, D.S., 2014. Carbon stock of the ecosystem of lower subtropical broadleaved evergreen forests of different ages in Pearl River Delta, China. Journal of Tropical Forest Science, pp.249-258.
82. Wang, G., Guan, D., Zhang, Q., et al., 2014. Spatial patterns of biomass and soil attributes in an estuarine mangrove forest (Yingluo Bay, South China). European Journal of Forest Research, 133(6), pp.993-1005.
83. Gurung, M.B., Bigsby, H., Cullen, R. et al., 2015. Estimation of carbon stock under different management regimes of tropical forest in the Terai Arc Landscape, Nepal. Forest Ecology and Management, 356, pp.144-152.
84. Hai, V.D., Do, T.V., Trieu, D.T., et al., 2015. Carbon stocks in tropical evergreen broadleaf forests in Central Highland, Vietnam. International Forestry Review, 17(1), pp.20-29.
85. Chheng, K., Sasaki, N., Mizoue, N., et al., 2016. Assessment of carbon stocks of semi-evergreen forests in Cambodia. Global Ecology and Conservation, 5, pp.34-47.
86. Ebasan, M.S., Aranico, E.C., Tampus, A.D. et al., 2016. Carbon stock assessment of three different forest covers in Panaon, Misamis Occidental, Philippines. Journal of Biodiversity and Environmental Sciences, 8(4), pp.252-264.
87. Fan, S., Guan, F., Xu, X., et al., 2016. Ecosystem carbon stock loss after land use change in subtropical forests in China. Forests, 7(7), p.142.
88. Mattsson, E., Ostwald, M., Wallin, G. et al., 2016. Heterogeneity and assessment uncertainties in forest characteristics and biomass carbon stocks: Important considerations for climate mitigation policies. Land use policy, 59, pp.84-94.
89. Pathak, R.P. and Baniya, C.B., 2016. Species Diversity and Tree Carbon Stock Pattern in a Community-Managed Tropical Shorea Forest in Nawalparasi, Nepal. International Journal of Ecology and Environmental Sciences, 42(S), pp.3-17.
90. Edwards, P.J. and Grubb, P.J., 1977. Studies of mineral cycling in a montane rain forest in New Guinea: I. The distribution of organic matter in the vegetation and soil. The Journal of Ecology, pp.943-969.
91. Kitayama, K. and Aiba, S.I., 2002. Ecosystem structure and productivity of tropical rain forests along altitudinal gradients with contrasting soil phosphorus pools on Mount Kinabalu, Borneo. Journal of Ecology, 90(1), pp.37-51.
92. Aiba, S.I., Takyu, M. and Kitayama, K., 2005. Dynamics, productivity and species richness of tropical rainforests along elevational and edaphic gradients on Mount Kinabalu, Borneo. Ecological Research, 20(3), pp.279-286.
93. Culmsee, H., Leuschner, C., Moser, G. et al., 2010. Forest aboveground biomass along an elevational transect in Sulawesi, Indonesia, and the role of Fagaceae in tropical montane rain forests. Journal of Biogeography, 37(5), pp.960-974.
94. Dossa, G.G., Paudel, E., Fujinuma, J., et al., 2013. Factors determining forest diversity and biomass on a tropical volcano, Mt. Rinjani, Lombok, Indonesia. PLoS One, 8(7), p.e67720.
95. Sawada, Y., Aiba, S.I., Seino, T. et al., 2016. Size structure, growth and regeneration of tropical conifers along a soil gradient related to altitude and geological substrates on Mount Kinabalu, Borneo. Plant and Soil, 403(1-2), pp.103-114.
96. Ma, L., Shen, C., Lou, D., et al., 2017. Patterns of ecosystem carbon density in edge-affected fengshui forests. Ecological Engineering, 107, pp.216-223.
97. Slik, J.W.F., Aiba, S.I., Brearley, F.Q., et al., 2010. Environmental correlates of tree biomass, basal area, wood specific gravity and stem density gradients in Borneo's tropical forests. Global Ecology and Biogeography, 19(1), pp.50-60.
98. Dai, W., Fu, W., Jiang, P., et al., 2018. Spatial pattern of carbon stocks in forest ecosystems of a typical subtropical region of southeastern China. Forest Ecology and Management, 409, pp.288-297.
99. Liu, X., Trogisch, S., He, J.-S., et al., 2018. Tree species richness increases ecosystem carbon storage in subtropical forests. Proceedings of the Royal Society B 285:20181240.
100. Nam, V.T., Anten, N.P.R. and van Kuijk, M., 2018. Biomass dynamics in a logged forest: the role of wood density. Journal of Plant Research, https://doi.org/10.1007/s10265-018-1042-9.
101. Wang, S., Zhuang, Q., Jia, S., et al., 2018. Spatial variations of soil organic carbon stocks in a coastal hilly area of China. Geoderma, 314, pp.8-19.
102. Mani, S. and Parthasarathy, N., 2007. Above-ground biomass estimation in ten tropical dry evergreen forest sites of peninsular India. Biomass & Bioenergy, 31(5), pp.284-290.
103. Pande, P.K. and Patra, A.K., 2010. Biomass and productivity in sal and miscellaneous forests of Satpura plateau (Madhya Pradesh) India. Advances in BioScience and Biotechnology, 1(1), pp.30-38.
104. Chaturvedi, R.K., Raghubanshi, A.S. and Singh, J.S., 2011. Carbon density and accumulation in woody species of tropical dry forest in India. Forest Ecology and Management, 262(8), pp.1576-1588.
105. Sundarapandian, S.M., Dar, J.A., Gandhi, D.S., et al., 2013. Estimation of biomass and carbon stocks in tropical dry forests in Sivagangai district, Tamil Nadu, India. International Journal of Environmental Science and Engineering Research, 4(3), pp.66-76.
106. Rabha, D., 2014. Aboveground Biomass and Carbon Stocks of an Undisturbed Regenerating Sal (*Shorea robusta* Gaertn. f.) Forest Of Goalpara District, Assam, Northeast India. International Journal of Environment, 3(4), pp.147-155.
107. Borah, M., Das, D., Kalita, J., et al., 2015. Tree species composition, biomass and carbon stocks in two tropical forest of Assam. Biomass & Bioenergy, 78, pp.25-35.
108. Lal, C., Singh, L., Sarvade, S. et al., 2016. Biomass and Carbon Storage Pattern in Natural and Plantation Forests of Sub-humid Tropics in Barnawapara Wildlife Sanctuary, Chhatisgarh, India. International Journal of Ecology and Environmental Sciences, 42(S), pp.83-90.
109. Majumdar, K., Choudhary, B.K. and Datta, B.K., 2016. Aboveground woody biomass, carbon stocks potential in selected tropical forest patches of Tripura, Northeast India. Open Journal of Ecology, 6, pp.598-612.
110. Salunkhe, O. and Khare, P.K., 2016. Aboveground Biomass and Carbon Stock of Tropical Deciduous Forest Ecosystems of Madhya Pradesh, India. International Journal of Ecology and Environmental Sciences, 42(S), pp.75-81.
111. Singh, V., Gupta, S.R. and Singh, N., 2016. Carbon Sequestration Potential of Tropical Dry Deciduous Forests in Southern Haryana, India. International Journal of Ecology and Environmental Sciences, 42(S), pp.51-64.
112. Ahirwal, J. and Maiti, S.K., 2017. Assessment of carbon sequestration potential of revegetated coal mine overburden dumps: a chronosequence study from dry tropical climate. Journal of Environmental Management, 201, pp.369-377.
113. Behera, S.K., Sahu, N., Mishra, A.K., et al., 2017. Aboveground biomass and carbon stock assessment in Indian tropical deciduous forest and relationship with stand structural attributes. Ecological Engineering, 99, pp.513-524.
114. Gogoi, A., Sahoo, U.K. and Singh, S.L., 2017. Assessment of Biomass and Total Carbon Stock in a Tropical Wet Evergreen Rainforest of Eastern Himalaya along a Disturbance Gradient. Journal of Plant Biology and Soil Health, 4(1):8.
115. Nath, S., Nath, A.J., Sileshi, G.W. et al., 2017. Biomass stocks and carbon storage in *Barringtonia acutangula* floodplain forests in North East India. Biomass & Bioenergy, 98, pp.37-42.
116. Niirou, N. and Gupta, A., 2017. Phytosociological analysis and carbon stocks for trees in different land uses in Senapati district of Manipur, India. Pleione, 11(1), pp.64-70.
117. Thokchom, A. and Yadava, P.S., 2017. Biomass and carbon stock along an altitudinal gradient in the forest of Manipur, Northeast India. Tropical Ecology, 58(2), pp.389-396.
118. Ramachandran, A., Jayakumar, S., Haroon, R.M., et al., 2007. Carbon sequestration: estimation of carbon stock in natural forests using geospatial technology in the Eastern Ghats of Tamil Nadu, India. Current Science, pp.323-331.
119. Mohanraj, R., Saravanan, J. and Dhanakumar, S., 2011. Carbon stock in Kolli forests, Eastern Ghats (India) with emphasis on aboveground biomass, litter, woody debris and soils. iForest-Biogeosciences and Forestry, 4, pp.61-65.
120. Sahu, S.C., Suresh, H.S. and Ravindranath, N.H., 2016. Forest structure, composition and above ground biomass of tree community in tropical dry forests of Eastern Ghats, India. Notulae Scientia Biologicae, 8(1), pp.125-133.
121. Gandhi, D.S. and Sundarapandian, S., 2017. Large-scale carbon stock assessment of woody vegetation in tropical dry deciduous forest of Sathanur reserve forest, Eastern Ghats, India. Environmental Monitoring and Assessment, 189(4), p.187.
122. Naveenkumar, J., Arunkumar, K.S. and Sundarapandian, S.M., 2017. Biomass and carbon stocks of a tropical dry forest of the Javadi Hills, Eastern Ghats, India. Carbon Management, pp.1-11.
123. Swamy, S.L., Dutt, C.B.S., Murthy, M.S.R., et al., 2010. Floristics and dry matter dynamics of tropical wet evergreen forests of Western Ghats, India. Current Science, 99(3), pp.353-364.
124. Murthy, I.K., Bhat, S., Sathyanarayan, V., et al., 2015. Biomass and Carbon Stock Dynamics in Tropical Evergreen and Deciduous Forests of Uttara Kannada District, Western Ghats, India. Global Journal of Science Frontier Research: H Environment & Earth Science, 15(5), https://globaljournals.org/item/5586-biomass-and-carbon-stock-dynamics-in-tropical-evergreen-and-deciduous-forests-of-uttara-kannada-district-western-ghats-india
125. Jeyakumar, S., Ayyappan, N., Muthuramkumar, S. et al., 2017. Impacts of selective logging on diversity, species composition and biomass of residual lowland dipterocarp forest in central Western Ghats, India. Tropical Ecology, 58(2), pp.315-330.
